# Effectiveness of pneumococcal vaccines in preventing pneumonia in adults, a systematic review and meta-analyses of observational studies

**DOI:** 10.1371/journal.pone.0177985

**Published:** 2017-05-23

**Authors:** Myint Tin Tin Htar, Anke L. Stuurman, Germano Ferreira, Cristiano Alicino, Kaatje Bollaerts, Chiara Paganino, Ralf René Reinert, Heinz-Josef Schmitt, Cecilia Trucchi, Thomas Vestraeten, Filippo Ansaldi

**Affiliations:** 1 Pfizer: Vaccines Clinical Epidemiology, Pfizer Inc, Paris, France; 2 P95 Epidemiology and Pharmacovigilance Consulting and Services, P95, Leuven, Belgium; 3 Department of Health Sciences (DiSSal), University of Genoa, Genoa, Italy; 4 Pfizer: Vaccines Medical Development and Scientific Clinical Affairs, Pfizer Inc, Paris, France; Universidade Nova de Lisboa Instituto de Higiene e Medicina Tropical, PORTUGAL

## Abstract

**Introduction:**

*S*. *pneumoniae* can cause a wide spectrum of diseases, including invasive pneumococcal disease and pneumonia. Two types of pneumococcal vaccines are indicated for use in adults: 23-valent pneumococcal polysaccharide vaccines (PPV23) and a 13-valent pneumococcal conjugate vaccine (PCV13).

**Objective:**

To systematically review the literature assessing pneumococcal vaccine effectiveness (VE) against community-acquired pneumonia (CAP) in adults among the general population, the immunocompromised and subjects with underlying risk factors in real-world settings.

**Methods:**

We searched for peer-reviewed observational studies published between 1980 and 2015 in Pubmed, SciELO or LILACS, with pneumococcal VE estimates against CAP, pneumococcal CAP or nonbacteremic pneumococcal CAP. Meta-analyses and meta-regression for VE against CAP requiring hospitalization in the general population was performed.

**Results:**

1159 unique articles were retrieved of which 33 were included. No studies evaluating PCV13 effectiveness were found. Wide ranges in PPV23 effectiveness estimates for any-CAP were observed among adults ≥65 years (-143% to 60%). The meta-analyzed VE estimate for any-CAP requiring hospitalization in the general population was 10.2% (95%CI: -12.6; 33.0). The meta-regression indicates that VE against any-CAP requiring hospitalization is significantly lower in studies with a maximum time since vaccination ≥60 months vs. <60 months and in countries with the pediatric PCV vaccine available on the private market. However, these results should be interpreted cautiously due to the high influence of two studies. The VE estimates for pneumococcal CAP hospitalization ranged from 32% (95%CI: -18; 61) to 51% (95%CI: 16; 71) in the general population.

**Conclusions:**

Wide ranges in PPV23 effectiveness estimates for any-CAP were observed, likely due to a great diversity of study populations, circulation of *S*. *pneumoniae* serotypes, coverage of pediatric pneumococcal vaccination, case definition and time since vaccination. Despite some evidence for short-term protection, effectiveness of PPV23 against CAP was not consistent in the general population, the immunocompromised and subjects with underlying risk factors.

## Introduction

*Streptococcus pneumoniae* can cause a wide spectrum of diseases, and is the leading cause of community-acquired pneumonia (CAP) [1]. In adults CAP is the most common type of pneumococcal disease [2]. Pneumococcal CAP (pCAP) can either be invasive bacteraemia or nonbacteremic (non-invasive). Two types of vaccines are indicated for use in adults in Europe: 23-valent pneumococcal polysaccharide vaccines (PPV23) [3, 4] first licensed in 1983, and a 13-valent pneumococcal conjugate vaccine (PCV13) which was first licensed in 2011 [3, 4].

There is evidence that pneumococcal vaccines are effective in preventing invasive pneumococcal disease (IPD) in adults. Several meta-analyses of randomised controlled trials (RCTs) have described clinical efficacy of PPV23 against IPD ranging from 10% to 80%. The clinical efficacy of PCV13 was 75% for vaccine-type IPD and 52% for any-cause IPD in adults aged 65 years or older in a recent RCT (CAPiTA trial)[5].

The absolute burden of vaccine preventable disease is much higher for CAP than for IPD [1, 6, 7]. The ability of pneumococcal vaccines to protect against CAP in adults is key in evaluating the overall public health benefits of the vaccination program. efficacy [8]. However, available clinical efficacy estimates of pneumococcal vaccines against CAP widely vary and are highly influenced by study settings and case definitions. The meta-analyzed clinical efficacy of PPV23 ranged from -11% to 74% for pCAP and from -13% to 29% for any-cause CAP [2, 9–11]; the clinical efficacy of PCV13 was 31% for pCAP and 5% for any-cause CAP [5].

A recent review estimated the real-world effectiveness of PPV23 for CAP among adults (>50 years) as 17% (95% CI: −26% to 45%) and 7% (95% CI: −10% to 21%) for cohort and case–control studies, respectively [11]. There was no real-world data available for PCV13 due its recent use in adults. To date, no systematic literature review and meta-analysis has been exclusively conducted for post-marketing observational studies evaluating the real-world effectiveness of pneumococcal vaccines against CAP in the general adult population, in immunocompromised and in immunocompetent patients.

We aimed to systematically review the literature on observational studies that assessed pneumococcal vaccine effectiveness (VE) against CAP (any-CAP, pCAP or nonbacteremic pCAP) in adults (see File 1 for PICO questions), with specific emphasis on any-CAP requiring hospitalization in the general population.

## Methods

### Identification of studies

MEDLINE (Pubmed), LILACS and SCIELO were searched to identify peer-reviewed articles published between January 1^st^ 1980 and October 30^th^ 2015 (date of search), in English, French, Spanish, Portuguese, Dutch, German and Italian. The search string consisted of terms for pneumococcal vaccines combined with terms for effectiveness (see S1 File for details). In addition, a grey literature search was performed through major public health organization websites and targeted search terms using Google. The protocol is available (S2 File).

### Inclusion criteria

We included published peer-reviewed observational studies conducted in adults (16 years or older), of any design (case-control, cohort, indirect cohort design (Broome method), test-negative case-control, and screening-method) of any pneumococcal vaccines indicated and used for adults (PPV23 or PCV13) that reported VE results on the protection for any clinically relevant outcome other than IPD; and that focused on direct effects [12]. Studies reporting exclusively on IPD and exclusively on the impact of pneumococcal vaccination program in adults were excluded. VE estimates comparing receipt of both PPV23 and influenza vaccine with the receipt of either influenza vaccine only or neither vaccine were excluded.

### Selection and data collection

Two reviewers (AS, GF) independently screened all titles and abstracts. The full-text of selected papers was reviewed and data were extracted (AS). For quality control results for 10% of the papers were extracted in duplicate (GF). An extensive hand search was conducted, based on the reference lists of relevant papers retrieved from the search, to identify additional studies. Data extraction was performed using MS Excel and Access. A complete list of data extraction items is available (S1 File).

### Clinical outcomes

Pneumonia was considered as community-acquired unless otherwise reported. CAP was classified into: any-CAP, pCAP, and nonbacteremic-pCAP. No a priori case definitions for CAP, pCAP or nonbacteremic-pCAP were set for this review, the case definitions used by the respective authors were accepted. Within each pneumonia type, estimates were further classified by healthcare setting (any setting, ambulatory, outpatient, hospitalized), and ‘mortality’.

### Effect measures

Estimates of VE expressed as percentage (%) as well as their confidence intervals (CIs) were derived from the effect measures, odds ratios, relative risks, hazard ratios and incidence rate ratios using VE = [1-effect measure]x100. VE estimates >0% suggest a protective effect.

### Quality assessment

Study limitations and risk of bias were assessed using the Newcastle-Ottawa assessment scale for cohort and case-control studies with scores ranging from 0 to 9 [13]. The following criteria were assessed: selection and comparability of study populations, and exposure (in case-control studies) or outcome (in cohort studies).

### Population

The studies were classified according to study population: general population, immunocompromised patients, and patients with underlying risk factors (other than immunosuppression). General populations consisted of populations from primary care centers, health maintenance organizations and insurance companies regardless of individual health status.

### Meta-analyses

We conducted meta-analyses for VE against any-CAP requiring hospitalization. Study heterogeneity was investigated by the chi-squared test for heterogeneity, for which p-values <0.05 indicate a significant amount of heterogeneity, and quantified using the I^2^ statistic with low, moderate and high levels of heterogeneity corresponding to I^2^ values of 25%, 50% and 75%, respectively. When I^2^ > 25%, a random-effects model was applied to obtain the pooled effect estimate [14].

Potential sources of study heterogeneity were explored by conducting stratified meta-analyses and by using meta-regression. The potential sources of heterogeneity we explored include: usage of pediatric pneumococcal vaccine (no PCV use, PCV available in private market, PCV in national immunization program), age (<65 years, ≥ 65 years, no specific age group described), study design (cohort or case-control) and maximum time since vaccination (<60 months, ≥ 60 months). The maximum time since vaccination refers to the greatest possible duration between the occurrence of the outcome and the vaccination by design of the study. R^2^ index was used to quantify the proportion of variance explained by the covariates in the meta-regression.

The influence of inclusion of a study on the results of the meta-analyses was assessed using study deletion diagnostics including Cook’s distance and DFBETAs. Publication bias was assessed visually using funnel plots. One additional post-hoc sensitivity analysis was carried out excluding all but the most recent of studies with potentially overlapping study populations. All analyses were performed in R version 3.2.0, using the metafor package for meta-analyses [15, 16].

## Results

**Fig 1:**
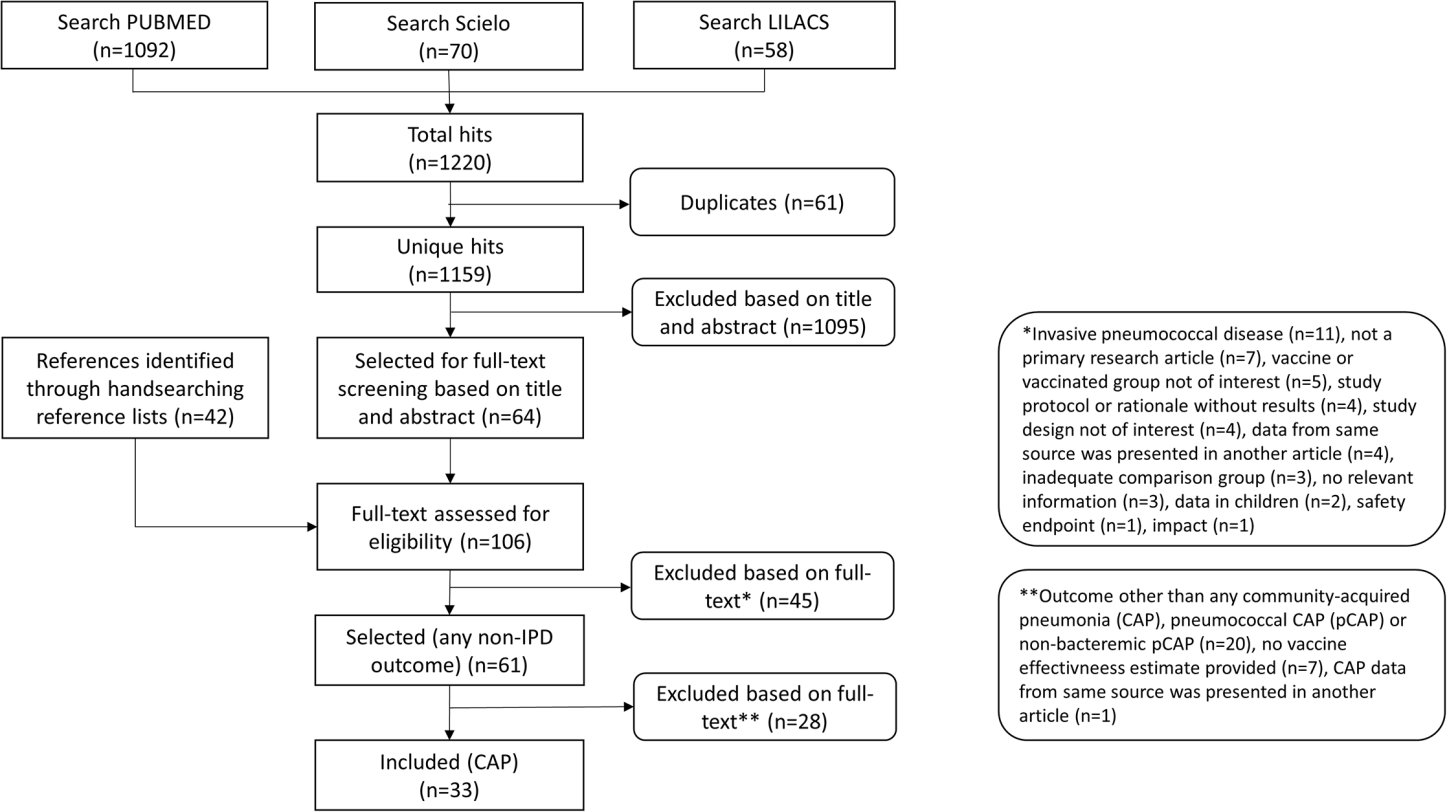
Flowchart of selection procedure. The flowchart was based on the flowchart from the PRISMA group [17]. *Invasive pneumococcal disease (n = 11), not a primary research article (n = 7), vaccine or vaccinated group not of interest (n = 5), study protocol or rationale without results (n = 4), study design not of interest (n = 4), data from same source was presented in another article (n = 4), inadequate comparison group (n = 3), no relevant information (n = 3), data in children (n = 2), safety endpoint (n = 1), impact (n = 1). **Outcome other than any community-acquired pneumonia (CAP), pneumococcal CAP (pCAP) or nonbacteremic pCAP (n = 18), no vaccine effectiveness estimate provided (n = 7), estimates comparing receipt of both PPV23 and influenza vaccine with the receipt of influenza vaccine only (n = 2), CAP data from same source was presented in another article (n = 1).

The search retrieved 1159 unique articles. Grey literature and public health websites searches retrieved no additional reports. After selection and hand search, 61 studies reported on VE against any clinically relevant outcome other than IPD in adults (Fig 1).

Of these, 33 studies on CAP reported at least one VE estimate. By study design, there were 20 cohort studies, 11 case-control studies, one case-cohort study and one study with self-controlled risk windows. By etiology 29 studies reported VE for any-CAP, 8 for pCAP and 5 for nonbacteremic-pCAP across all study populations. No studies reported effectiveness of PCV13 in adults. Newcastle-Ottawa quality scores ranged from 4 to 7 and from 3 to 8 for cohort and for case-control studies (S1 Table).

### Any-CAP

Overall, 15 studies reported VE estimates for CAP in the general population, 9 in the immunocompromised, and 8 in patients with underlying risk factors. 7 studies were conducted by one research group in primary care centers in Tarragona (Spain) and therefore overlapping study populations cannot be excluded. Estimates with detailed study description are reported in S2 Table.

#### Any-CAP in general population

**Fig 2:**
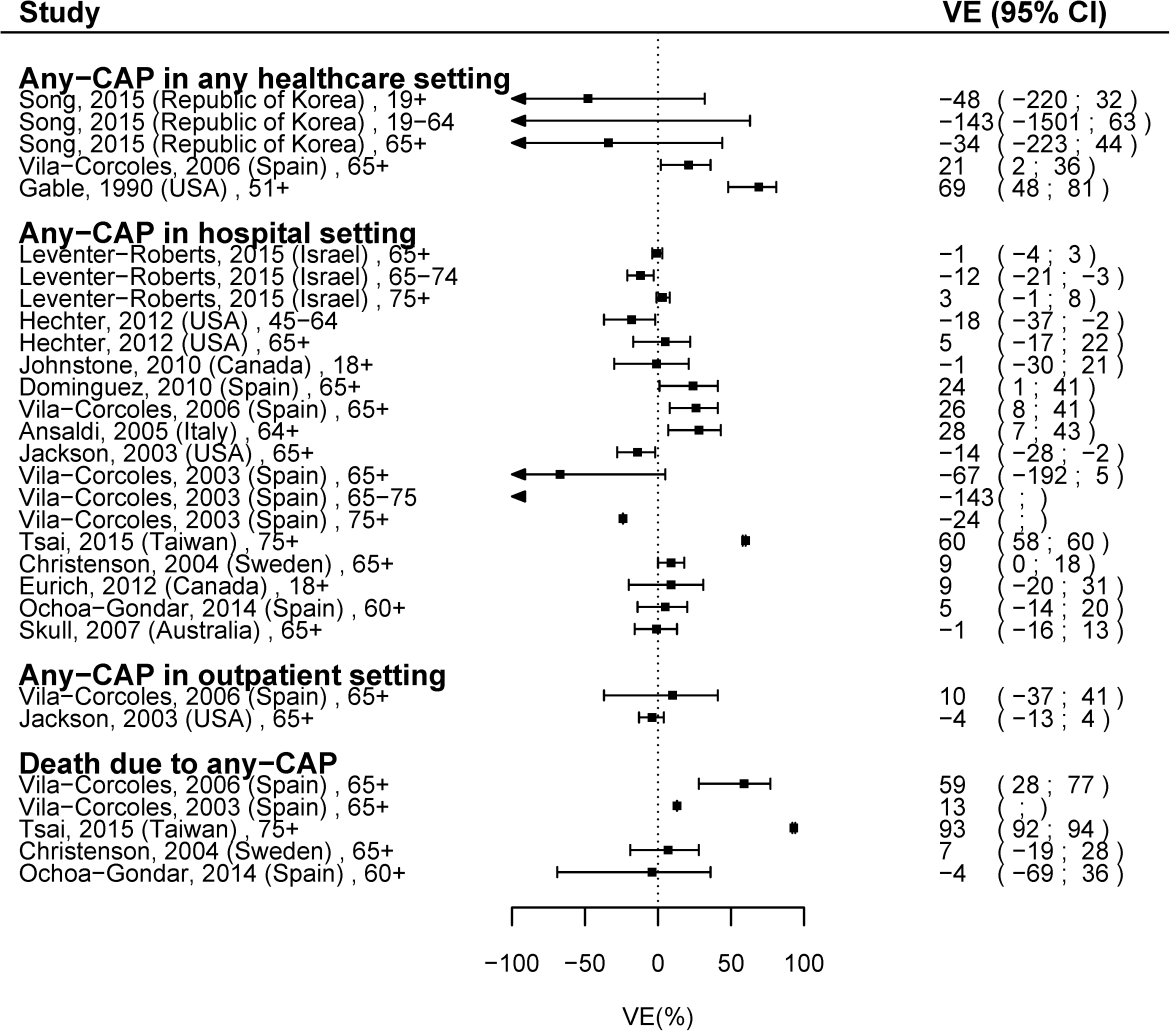
VE with most adjusted estimates for any-CAP in the general population, comparing PPV23 vaccinated with unvaccinated.

For protection against CAP hospitalization in the general population, there were 13 studies and 18 VE estimates (Fig 2). Several studies were conducted in Spain (n = 4) for which most estimates only included adults >65 years. Among adults aged >65 years, the VE estimates ranged from -143% [18] to 60% [19].

The overall meta-analyzed VE for any-CAP hospitalization was 10.20% (95%CI -12.62; 33.01). The between-study heterogeneity was high (I^2^ = 99.24%, p < 0.01). The study by Tsai [19] was found to be influential (DFBETA = 1.46). After exclusion of this study, the meta-analyzed VE was 5.14% (95%CI: -2.09; 12.37, I^2^ = 71.66%).

Based on the meta-regression, usage of pediatric pneumococcal vaccine and maximum time since vaccination were both significant (p<0.01) and explained part of the heterogeneity (R^2^ = 77.4%; I^2^ = 41.2%;) (S3 Table). However, these results should be interpreted with caution since two studies (Tsai et al. [19] and Christenson et al. [20]) were found to be influential.

**Fig 3:**
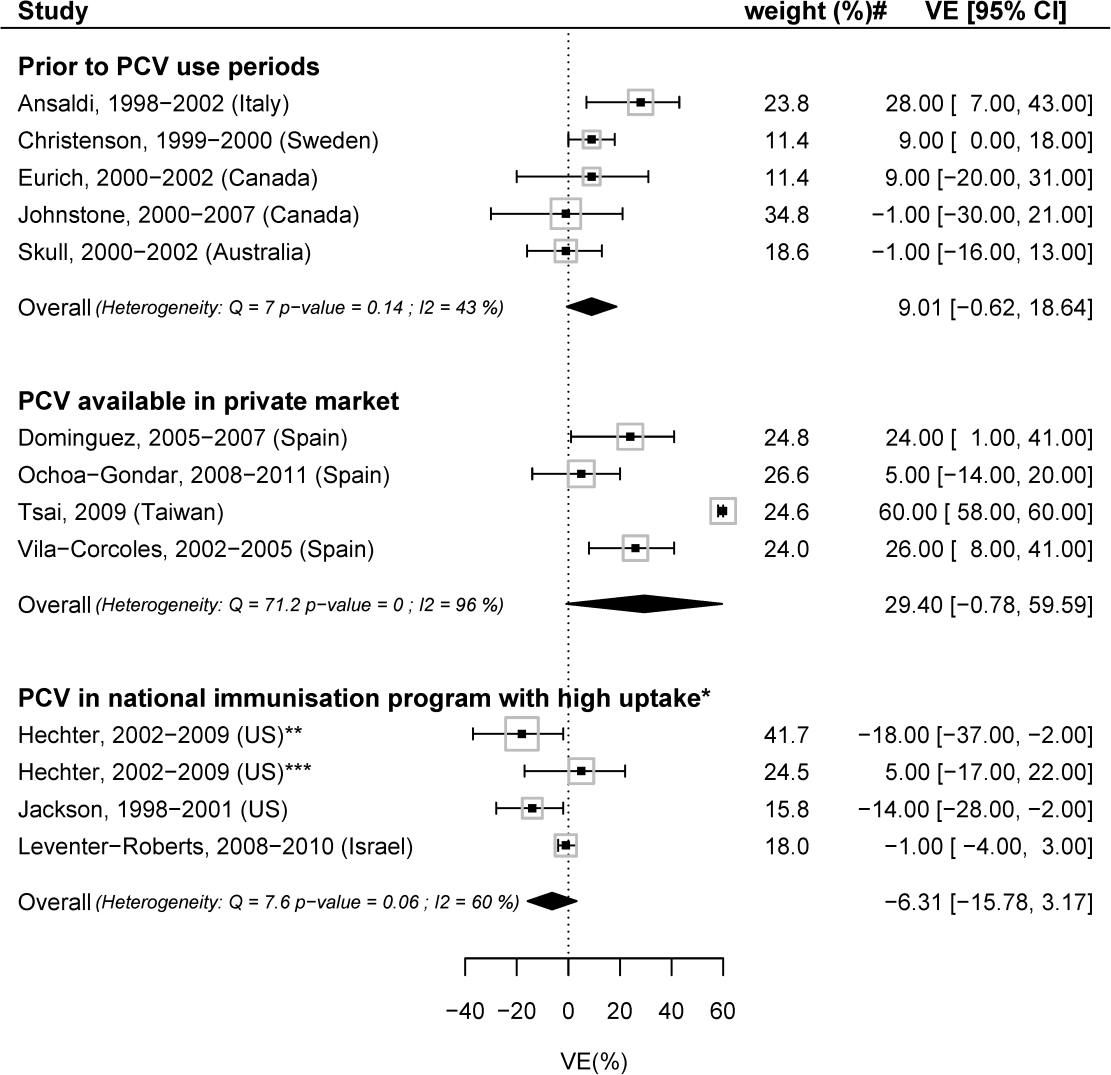
Meta-analysis, stratified by availability of pediatric pneumococcal vaccine (PCV): VE with most adjusted estimates for hospitalization due to any-CAP in the general population, comparing PPV23 vaccinated with unvaccinated. # weights from the random-effects model; *>70% for at least one dose; **45–64 years; ***65+ years.

**Fig 4:**
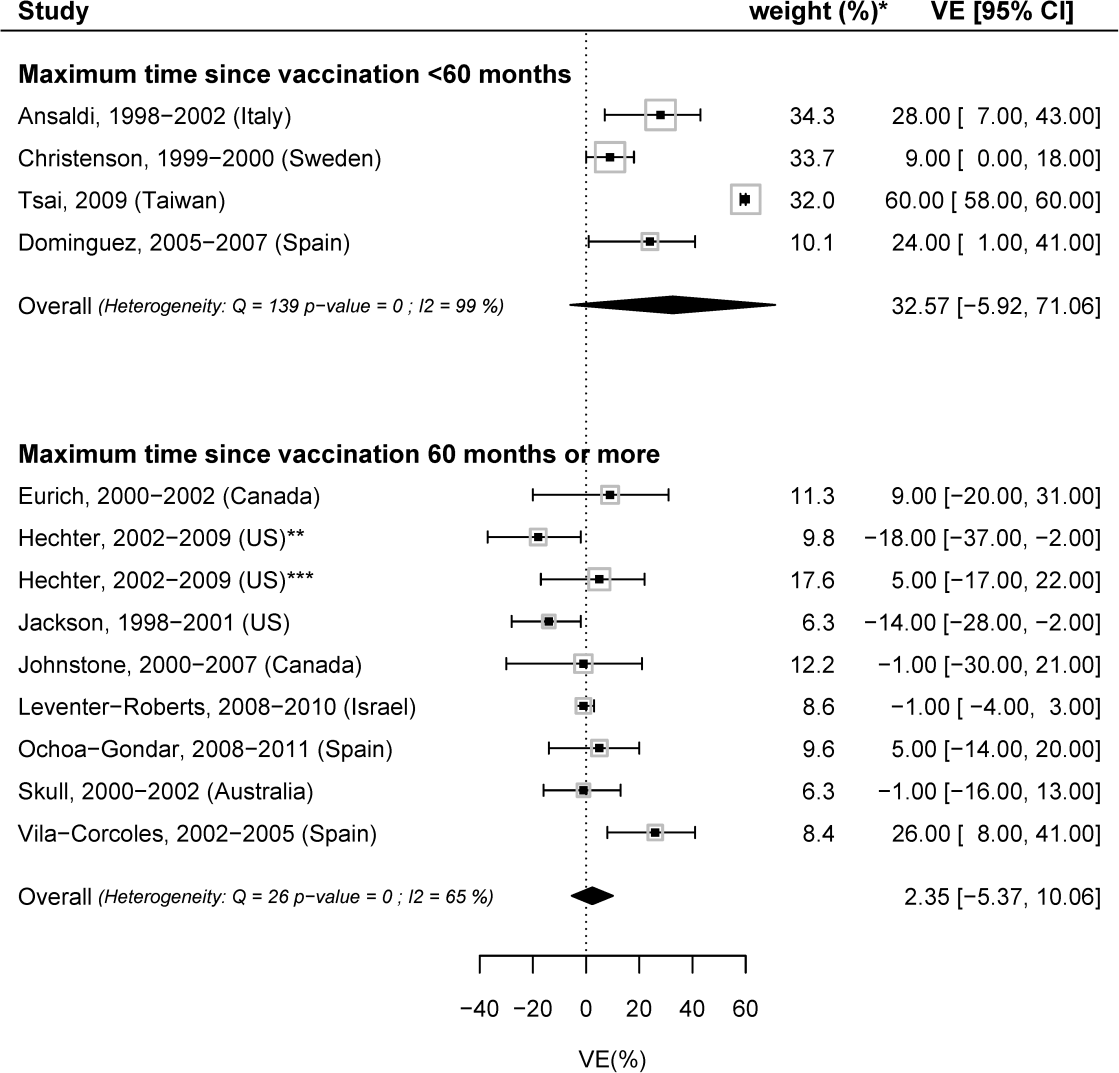
Meta-analysis, stratified by maximum time since vaccination: VE with most adjusted estimates for hospitalization due to any-CAP in the general population, comparing PPV23 vaccinated with unvaccinated. *weights from the random-effects model; **45–64 years; ***65+ years.

In the stratified meta-analysis by pediatric PCV use, the meta-analyzed VE was -6.31 (95%CI: -15.78; 3.17, I^2^ = 60%,), 9.01 (95%CI: −0.62; 18.64, I^2^ = 43%) and 29.40 (95%CI: -0.78; 59.59, I^2^ = 96%) when pediatric PCV was part of the national immunization program, when pediatric PCV was not available and when PCV was available in the private market only (Fig 3). In the stratified meta-analysis by maximum time since vaccination, the meta-analyzed VE was 32.6% (95%CI: -5.9; 71.1, I^2^ = 99%) and 2.4% (95%CI: -5.4; 10.1, I^2^ = 65%) when the time since vaccination was less than 60 months and 60 months or more, respectively. (Fig 4). There was no evidence of publication bias.

Among three studies evaluating VE against any-CAP in any healthcare setting, VE ranged from -143% (95%CI: -1501; 63) [21] to 69% (95%CI: 48; 81) [22]. Neither of two studies presenting VE for any-CAP in outpatient setting was statistically significant [23, 24]. Among 5 studies evaluating VE against death due to any-CAP, estimates ranged from -4% (95% CI: -69; 36)[25] to 93% (95% CI: 92; 94) [19].

#### Any-CAP in immunocompromised patients and patients with underlying risk factors

S1 and S2 Figs summarize the VE estimates for any-CAP in immunocompromised populations and populations with underlying risk factors, respectively.

Among 9 studies in immunocompromised patients VE estimates ranged from -88% (95% CI: -349; 21) [26] to 78% (95%CI: 46; 91) [27] across age groups, CD4 counts, severity and healthcare settings. VE against any-CAP in HIV-infected individuals decreased with increasing viral load [28], but no effect of CD4 count was observed [28, 29].

Among 8 studies among patients with underlying risk factors, the VE against any-CAP ranged from -338% [18] to 72% [30] (S2 Fig). Four studies were conducted in patients with chronic respiratory and/or COPD aged 65 years or older [31–34]. These yielded 7 VE estimates, of which only two reached statistical significance [31, 33]. Neither of the two VE estimates against death due to any-CAP was statistically significant [30, 31].

### Pneumococcal CAP

There were 8 studies evaluating the VE of pCAP, five studies were from Tarragona region, Spain (S4 Table). For pCAP, the identification of etiologic agent was made from blood or other sterile site culture, sputum or other respiratory samples culture, or urinary antigen test or from clinical diagnosis codes. The proportion of bacteremic pCAP cases among all pCAP cases varied from 11 [25] to 33% [35, 36] in the general population, 58% in the HIV-infected adults study [37] and 20% in the study conducted in chronic respiratory disease patients [38].

#### Pneumococcal CAP in general population

Four studies evaluated the VE for pCAP in the general population. All four studies were in subjects aged 50 years or older and three were conducted in Tarragona. The VE estimates for pCAP irrespective of severity and setting were 45% (95%CI: 12; 66) [36] and 53% (95%CI: 33; 68) [35] among those aged 65 years or older. The estimates were slightly higher during the influenza season (60% and 61%) than out of the influenza season (49% and 37%) [35, 36]. The VE for hospitalization due to pCAP varied from 32% (95 CI: -18; 61) to 51% (95% CI: 16; 71) [25].

#### Pneumococcal CAP in immunocompromised patients and patients with underlying risk factors

Among two studies in immunocompromised patients, the VE estimates for pCAP were 77% (95%: -6; 92) in HIV-infected patients [37] and 71% (95%CI: 24; 89) in possibly immunocompromised patients (all causes combined) [35]. Among 4 studies identified in patients with underlying risk factors, the VE estimate for pCAP irrespective of severity and setting was 29% (95%CI: -39; 63) among those aged 50 years or older with chronic respiratory disease [38] and 41% (95%CI: 10; 61) for those with various chronic disease [35]; VE for pCAP hospitalization among those aged 65 years or older was 24% (95%CI: -90; 70) among those with chronic respiratory disease [31] and 38% (95%CI: -5; 70) among those with various risk factors [39]. None of VE estimates in patients with chronic respiratory disease reached statistical significance. The VE point estimates in patients with chronic respiratory diseases was lower outside the influenza season (versus influenza season), albeit statistically non-significant.

### Nonbacteremic-pCAP

Out of four identified studies conducted in the general population, three studies were from the same research group and geographical area in Tarragona, Spain. Study characteristics and VE estimates for nonbacteremic pCAP are presented in S5 Table. In these studies, potential CAP cases were initially identified based on ICD-9 codes with different case definitions for each study. Nonbacteremic-pCAP was defined when CAP was accompanied by a positive sputum culture for *S*. *pneumoniae* (but negative/not performed blood culture) and/or a positive Binax-NOW urinary antigen test [25, 35, 40], or only a positive Binax-NOW urinary antigen test [36]. In addition, two of these studies also used laboratory records to identify cases missed by ICD codes [35, 36]. One study in the US based the definition on a typical clinical syndrome of pneumonia and radiographic confirmation, sputum culture positive and at least one negative blood culture result for *S*. *peumoniae* [41].

The VE estimates for nonbacteremic-CAP of any severity or setting in the general population aged 50 years or older, ranged from 39% (95% CI: -6; 65) [42] to 42% (95%CI: 14; 61) [35]. The VE was -1% (95% CI: -86; 45) for nonbacteremic-pCAP in the US adult (18 years or older) study [41] irrespective of severity and setting and -3% (95% CI: -53; 31) for nonbacteremic-pCAP hospitalization in Spanish study [25]. The VE was higher (54%) during the influenza season compared to outside the influenza season (44%) [35].

No studies in immunocompromised patients were identified and only one study in patients with chronic respiratory disease was reported. The VE estimate was 34% (95% CI: -34; 67) [38].

### Vaccine effectiveness and time since vaccination

In general, lower VE was observed in studies with longer maximum time since vaccination (at least 60 months post-vaccination) [28, 35]. A Spanish prospective cohort study (CAPAMIS) showed how VEs varied according to the time since vaccination [25]. The VE increased and became more significant after excluding the patients vaccinated more than 60 months ago from the analysis. The VE estimates were 25% (95%CI: 2; 42), 51% (95% CI: 16; 71), 62% (95% CI: -68; 91) and 48% (95% CI: 8;71) in the analysis exclusively including the recently vaccinated patients (less than 60 months before study start, compared to those never vaccinated) for any-CAP, pCAP, bacteremic-pCAP and nonbacteremic-pCAP, respectively. The VE estimates were 8% (95%CI: -20; 29), 32% (95%CI: -18; 61), 53% (95% CI: -145; 91) and 29% (95% CI: -27; 61) for any-CAP, pCAP, bacteremic-pCAP and nonbacteremic-pCAP, respectively, in the analysis regardless of time since vaccination (i.e. comparing those vaccinated any time before study start to those never vaccinated).

### Sensitivity analysis

Since three out of 13 studies were conducted in health care centers from Tarragona region in Spain, a sensitivity analysis was carried out excluding all but the most recent of studies with potentially overlapping study populations. Particularly, the meta-analyses were repeated excluding Dominguez et al. 2010 [43] and Vila-Corcoles et al. 2006 [36, 44]. The overall meta-analyzed VE for any-CAP hospitalization was 7.52% (95%CI -17.71; 32.76, I^2^ = 99.36%).

## Discussion

This literature review exclusively describes pneumococcal VE in protecting against overall CAP in adults using observational studies. No studies reported effectiveness of PCV13 in adults, likely due to the recent PCV13 introduction and recommendation for adult population. Analyses were first stratified by populations (general population, immunocompromised patients and patients with underlying risk factors) and health care setting. Although availability of pediatric vaccine and time since vaccination partially explained between-study heterogeneity, the remaining heterogeneity remained substantial. Indeed, the included studies were heterogeneous in different aspects, including study populations, case definitions, case ascertainment, healthcare practices and pneumococcal serotype epidemiology, in particular variations in distribution of serotypes over time and across regions.

A wide range of VE estimates for any-CAP in adults was found, regardless of population type and healthcare setting. Overall, PPV23 was not consistently demonstrated to be effective in protecting against any-CAP in the general population, although some evidence for short-term vaccine effectiveness exists. In fact, the meta-analyzed VE for hospitalized CAP in the general population was 10.20% (95%CI -12.62; 33.01). The VE among the general population was lower than the meta-analyzed VE estimated from RCTs, 27% (95%CI: 6; 44) and 28% (95% CI: 7; 44) in two meta-analyses [2, 9], and higher than -10% (95% CI: -30; 7) in a recent meta-analysis [45]. On the other hand, our results are similar to another review based on observational studies with VEs of 17% (95%CI: -26; 45) and 7% (95%CI: -10; 21) for cohort and case-control studies respectively [11].

The VE of PPV23 for any-CAP in immunocompromised patients or patients with underlying risk factors (COPD and chronic respiratory disease) remains controversial. In our review, the VE ranged from -88% to 78% in immunocompromised patients (mostly HIV-infected patients) and from -338% to 72% in patients with underlying risk factors. These findings confirmed those obtained from meta-analyses of RCTs conducted among these patients [2, 9–11].

As expected, VE estimates for pCAP in all populations were consistently higher than against any-CAP [25, 31, 35–39, 46]. This review revealed positive VE estimates for pCAP, 45% and 53% in adults aged 65 years or older in the general population [35, 36]. Our VE estimates for nonbacteremic pCAP ranged from 39 to 42% in adults aged 50 years or older in the general population. In meta-analyses of RCTs, pCAP is often split into presumptive pCAP (nonbacteremic) and definite pCAP (bacteremic). Our findings for nonbacteremic pCAP were similar to two meta-analyzed efficacy estimates for presumed pCAP based on RCTs, 54% (95% CI: 16; 75) and 36% (4; 57) [2, 9].

The PPV23 effectiveness depends on time since vaccination. In the CAPAMIS study, the effectiveness estimates became higher and significant after excluding the patients vaccinated for more than 60 months ago [25]. Our meta-analyzed VE was 2% (95%CI: -5;10) in studies where the maximum time since vaccination was 60 months or more while it was 33% (95%CI: -6; 71) in patients vaccinated less than 60 months ago. These results are consistent with immunological findings where the initial rise in antibody titers declined over time, reaching to approximate pre-vaccination baseline after 4–7 years of vaccination [47, 48]. The lack of efficient memory induction by PPV23 would explain these waning protection [49].

Specific precautions are necessary to interpret the effect of pneumococcal vaccine in adults in populations with different approaches to pediatric pneumococcal vaccination. In the US and Israel, the pediatric PCV uptake was high and this likely led to reduce vaccine-serotype *S*. *pneumoniae* circulation, which in turn could have led to a lower proportion of vaccine serotype cases among any-CAP cases in the general population (herd protection), resulting in a lower VE estimate [17, 50]. Simultaneously, all studies in the category “PCV in national immunization program” also had a maximum time since vaccination of more than 60 months, likely lowering the VE estimate further. The effectiveness estimate in settings with no PCV was positive and higher than the estimate for “PCV in national immunization program”, and the estimate was higher still in settings with “PCV available in private market”. It is expected that the estimate for “PCV available in private market” (with low PCV uptake at the time of the study) is higher than that for “PCV in national immunization program” (with high PCV uptake). However, it is counterintuitive that the estimate for “PCV available in private market” is higher than the one for settings with no PCV; this warrants further research that takes into account detailed information on factors such asthe actual pediatric vaccination coverage and time since vaccination.

### Limitations

We restricted the meta-analysis to any-CAP requiring hospitalization in the general population due to the limited number of studies reporting on the other outcomes.

Heterogeneity in case ascertainment and case definitions between the studies would influence the final results. CAP diagnosis was sometimes based on ICD-9 codes only (e.g. ICD-9 480–487 [51] or ICD-9 codes 481, 486, 482.9, 485, V1261 [52]); on ICD-9 codes complemented by a medical record check and a chest radiograph for confirmation [36]; or on signs and symptoms and a chest radiograph [43]. However, almost all studies required radiographic and laboratory-confirmation for a diagnosis of pCAP (including nonbacteremic pCAP).

A further potential source of bias may be the misclassification of invasive diseases as non-invasive diseases. In studies from Tarragona, non-bacteremic pCAP was defined as clinical and radiological pneumonia with negative blood culture and/or not performed blood culture, and sputum culture positive for *S*. *pneumonia* and/or positive Binax [35, 40]. However, the proportion of patients with non-performed culture and prior antibiotics before the diagnosis was unknown. Moreover, most pCAP diagnoses in the observational studies involved identification of *S*. *pneumonia* from either sterile or non-sterile site culture. Including isolates from sterile culture in the definition is a possible explanation for the fact that VE for pCAP in observational studies is close to VE for IPD in RCTs. Heterogeneity can exist in the extent of misclassification between the studies. Nonetheless, the proportion of invasive diseases among pCAP was known for few studies [35–38].

The definition of maximum time since vaccination differed between studies and could have introduced additional heterogeneity. The meta-analysis and meta-regression of results stratified by maximum time since vaccination attempted to partially overcome this limitation.

Although the most adjusted estimate was considered, and many studies adjusted for the effect of influenza vaccination, this was not always taken into account. The influenza vaccine is known to be effective in reduction of any-CAP and hence, combined vaccination with pneumococcal vaccine might overestimate the VE attributed to pneumococcal vaccine in the general population [53, 54].

An inherent limitation of observational studies is that vaccines are channeled to certain populations. For example, frailer patients (e.g. older, more co-morbidities) with a higher baseline risk for CAP, hospitalization and death may be more likely to be targeted for vaccination, therefore possibly underestimating the effect of pneumococcal vaccines. Simultaneously, the healthy vaccinee effect could result in an overestimation of the VE. To minimize the influence of these limitations, most included studies adjusted for the potential confounders using propensity scores or multiple regression between vaccinated and non-vaccinated patients.

Study quality was assessed using the Newcastle Ottawa score. While important aspects of study design are rated using this tool, it did not take into account the rationale and the context (e.g. local *S*. *pneumoniae* epidemiology, healthcare practices) in which a study was initiated and performed. Missing information in the publication lowers the overall score.

## Conclusions

Wide ranges in PPV23 effectiveness estimates for any-CAP were observed, with a higher, albeit not statistically significantly, effectiveness in more recently vaccinated populations and in settings with low-to-moderate paediatric PCV vaccine uptake. Overall, the effectiveness of PPV23 against CAP has not been consistently demonstrated in the general population (including the elderly), the immunocompromised and subjects with underlying risk factors. This lack of consistency may be related to a great diversity of study populations, circulation of *S*. *pneumoniae* serotypes, coverage of pneumococcal pediatric vaccination.

Pneumococcal vaccination programs, both adult and pediatric programs as an integrated public health intervention are likely to impact the proportion of CAP caused by *S*. *pneumoniae* and thus influencing the effectiveness of the vaccines evaluated. Monitoring of adult pneumococcal VE should continue in the context of increasing PCV13 use in adults and increasing vaccination coverage in the pediatric population.

## Supporting information

S1 File Methods. (DOCX)

S2 File Protocol. (PDF)

S3 File Prisma checklist. (DOCX)

S4 File References for all included studies. (DOCX)

S1 Fig VE with most adjusted estimates for any-CAP in immunocompromised populations, comparing PPV23 (or other Pneumococcal vaccine NOS) vaccinated with unvaccinated. (TIF)

S2 Fig VE with most adjusted estimates for any-CAP in patients with underlying risk factors, comparing PPV23 (or other Pneumococcal vaccine NOS) vaccinated with unvaccinated. (TIF)

S1 Table Quality assessment of included studies using the Newcastle-Ottawa Score. (DOCX)

S2 Table Study characteristics and results for any-CAP. (DOCX)

S3 Table Results of the meta-regression: Vaccine effectiveness against CAP requiring hospitalization. (DOCX)

S4 Table Study characteristics and results for pneumococcal CAP. (DOCX)

S5 Table Study characteristics and results for nonbacteremic pneumococcal CAP. (DOCX)
