# Supplementary material for: Effectiveness of pneumococcal vaccines in preventing pneumonia in adults, a systematic review and meta-analyses of observational studies
Source: PLoS One. 2017 May 23;12(5):e0177985. doi: 10.1371/journal.pone.0177985 (PMC5441633; doi:10.1371/journal.pone.0177985)
Supplement: S1 File — (DOCX) [file pone.0177985.s001.docx]

**Methods**

*PICO question*

Table A. PICO for main research question

|  | What is the vaccine effectiveness of pneumococcal vaccines in adults? |
| --- | --- |
| P | Adults |
| I | Pneumococcal vaccine |
| C | No pneumococcal vaccine |
| O | CAP (any-CAP, pneumococcal CAP, nonbacteremic pneumococcal CAP) |

CAP: community acquired pneumonia

*Search string*

The search string used in Pubmed was: (Pneumococcal Vaccines[MeSH] OR (Pneumococc*[tiab] AND (vaccin* or immuniz*[tiab] OR immunis*[tiab]))) OR (PPV23[tiab] OR “PPV 23”[tiab] OR Pneumovax[tiab] OR PCV13[tiab] OR “PCV 13”[tiab] OR Prevnar[tiab] OR Prevenar[tiab] OR "Pneumo 23"[tiab] OR Pneumo23[tiab]) OR (Vaccination[Mesh] AND pneumococcal infections[Mesh]) AND (effectiveness[ti] OR effectiveness[ot] OR efficacy[ti] OR efficacy[ot] OR impact[ti] OR impact[ot] OR “treatment outcome”[mesh]). In addition, we conducted a grey literature search on public health websites.

We excluded studies that did not fulfill the above criteria, that did not present primary data, studies restricted to children, randomized controlled trials, economic evaluations, transmission modelling studies, studies that focused exclusively on invasive pneumococcal disease, studies on *S. pneumoniae* asymptomatic carriages, studies on immunogenicity and studies with non-human data. We also excluded conference abstracts.

*Extracted data items*

Data for the following items was extracted into an Excel file: Aim, country, region, vaccine type, study design, prospective/retrospective, study population, minimum age at inclusion, summary age in population, study period, setting, clinical outcome, case ascertainment methods, case definition, clinical outcome severity, definition of exposed, time since vaccination, definition of non-exposed, total study sample size, sample size by stratum (e.g. by age group), effect measure (point estimate, 95%CI), factors that were adjusted for, co-administration of vaccines (especially influenza), *S. pneumonia* serotypes, uptake/coverage for pneumococcal vaccines in the general population. Additionally, the following items were extracted for case-control studies: selection of controls, definition of control, number of cases, controls, cases who were exposed, controls who were exposed; and for cohort studies: number of exposed, non-exposed, cases, exposed cases, non-exposed cases, duration of follow-up.

*Description of immunocompromised populations and populations with underlying risk factors*

Immunocompromised populations included HIV-infected populations (regardless of CD4 count or viral load), end-stage renal disease patients on dialysis, and other immunocompromised patients (e.g. patients with systemic autoimmune disease on rituximab). Populations with underlying risk factors including among others patients with chronic respiratory disease, such as chronic obstructive pulmonary disease (COPD), those at high risk for myocardial infarction, smokers, hospitalized patients, geriatric hospital residents, patients at different risk levels for pneumonia, and patients with various risk factors not specified in the publication.
