# Supplementary material for: Effectiveness of pneumococcal vaccines in preventing pneumonia in adults, a systematic review and meta-analyses of observational studies
Source: PLoS One. 2017 May 23;12(5):e0177985. doi: 10.1371/journal.pone.0177985 (PMC5441633; doi:10.1371/journal.pone.0177985)
Supplement: S2 File — (PDF) [file pone.0177985.s002.pdf]

Systematic literature review and meta-analysis on the impact and effectiveness of pneumococcal vaccines against non-IPD in adults

Protocol

Version 2.0

Germano Ferreira
Marc Baay
Anke Stuurman
Kaatje Bollaerts
Thomas Verstraeten

Version	Date	Previous version	Changes made since previous number
2.0	20151113	1	Protocol updated after meeting with Pfizer
1.0	20151019	0	Protocol draft from P95 for Pfizer comments
0	20151001	NA	Protocol draft development started

Table of Contents

Table of Contents 2

1 Background 4

2 Objectives 5

3 Methods 5
- 3.1 Eligibility criteria 5
  - 3.1.1 Inclusion criteria 5
  - 3.1.2 Exclusion criteria 6
- 3.2 Information sources 6
  - 3.2.1 Electronic databases and search strategy 6
  - 3.2.2 Grey literature selection 6
- 3.3 Data collection and analysis 6
  - 3.3.1 Data management and storage 6
  - 3.3.2 Selection of studies 7
  - 3.3.3 Reference checking and hand searching 7
  - 3.3.4 Data collection 7
- 3.4 Data items 7
- 3.5 Outcomes 8
  - 3.5.1 Clinical outcomes 8
  - 3.5.2 Effect measures 8
- 3.6 Quality assessment 8

4 Analyses and reporting 9

5 Meta-analysis 9
- 5.1 Decision criteria 9
- 5.2 Analysis 9

6 References 9

Annex 1. Search strategy 11

Annex 2. Grey literature search 17

Annex 3. Risk of bias tools 18

Acronyms

CAPITA	Community-Acquired Pneumonia Immunization Trial in Adults
CDC	Centers for Disease Control and Prevention
COPD	chronic obstructive pulmonary disease
ECDC	European Centre for Disease Prevention and Control
GRADE	Grading of Recommendations Assessment, Development and Evaluation.
HIV	human immunodeficiency virus
HR	hazard ratio
InVS	Institut de veille sanitaire
INSPQ	Institut national de santé publique Québec
IPD	invasive pneumococcal disease
IRR	incidence rate ratio
LILACS	Latin American and Caribbean Health Sciences Literature
MI	myocardial infarction
PCV 13	pneumococcal conjugate vaccine 13 valences
PPV 23	pneumococcal polysaccharide vaccine 23 valences
PRISMA	Preferred Reporting Items for Systematic Reviews and Meta-Analyses
OR	odds ratio
RKI	Robert Koch Institute
RR	relative risk
SCIELO	Scientific Electronic Library Online
UK	United Kingdom
US	United States
VE	vaccine effectiveness
WHO	World Health Organization

1 Background

Streptococcus pneumoniae is an encapsulated Gram-positive coccus. The capsule is the most important virulence factor of *S. pneumoniae*; pneumococci that lack the capsule are normally not virulent. Over 90 different capsular types have been characterized, based on serology (1). In general, immunity following infection is serotype-specific, but cross-protection between related serotypes can occur (2).

Transmission is by aerosol, droplets or direct contact with respiratory secretions of someone carrying the organism. Transmission usually requires either frequent or prolonged close contact. There is a seasonal variation in pneumococcal disease, with peak levels in the winter months (1).

Pneumococcal infection and disease can affect a variety of organ systems resulting in a number of disease syndromes. Although temporary colonization of the nasopharyngeal mucosa, which is the primary focus of infection, rarely results in disease, certain pneumococcal serotypes may occasionally invade the blood stream causing bacteremia and possibly infection of secondary sites such as the meninges (meningitis). In other instances, contiguous spread from the nasopharynx may cause diseases such as otitis media or sinusitis. Pneumonia is often caused by aspiration of pneumococci from the nasopharynx. When associated with bacteremia, pneumonia is classified as invasive pneumococcal disease (2).

Currently, two vaccines are indicated for use in adults. The pneumococcal polysaccharide vaccine 23 (PPV23, trade name Pneumovax23, Merck - Sanofi Pasteur MSD in Europe) is a vaccine indicated for active immunization, as a single intramuscular or subcutaneous dose, for the prevention of pneumococcal disease caused by the 23 serotypes contained in the vaccine (1, 2, 3, 4, 5, 6B, 7F, 8, 9N, 9V, 10A, 11A, 12F, 14, 15B, 17F, 18C, 19F, 19A, 20, 22F, 23F, and 33F). PPV23 is approved for use in persons 50 years of age or older and persons aged ≥2 years who are at increased risk for pneumococcal disease (3). The vaccine does not protect against pneumococcal infection due to capsular types not contained in the vaccine, but the 23 types included account for about 96% of the pneumococcal isolates that cause serious infection in the UK, although serotype replacement has been described (1). PPV23 is considered safe both in terms of severe immediate reactions and potential long-term adverse consequences (2).

The other vaccine is the pneumococcal conjugate vaccine 13 (PCV13, tradename Prevenar 13, Pfizer), which is indicated for active immunization of adults, as a single intramuscular dose, for the prevention of pneumococcal disease caused by *S. pneumoniae* serotypes 1, 3, 4, 5, 6A, 6B, 7F, 9V, 14, 18C, 19A, 19F, and 23F (4). In some countries, its use is recommended for immunocompromised adults aged 19 years or older, including patients with functional or anatomic asplenia, HIV infection, cancer, advanced kidney disease, or other immunocompromising conditions (1).

Pfizer has requested P-95 to carry out a systematic review of the literature and a meta-analysis of observational studies to determine the impact and effectiveness of pneumococcal vaccines in preventing pneumococcal non-invasive disease, pneumococcal pneumonia, all-cause pneumonia, and all-cause diseases in adults.

2 Objectives

The objectives of this project are:

1. To perform a systematic literature review of the available published peer-reviewed and grey literature reports on observational studies assessing the
   a. Effectiveness of pneumococcal vaccines used in adults, and
   b. Impact of adult pneumococcal vaccination programs

   in preventing pneumococcal non-invasive disease, pneumococcal pneumonia, all-cause pneumonia, and any-cause diseases (including sequellae/complications of pneumonia).
2. To perform a meta-analysis, dependent on the availability and quality of estimates that would allow for a meta-analysis.

3 Methods

3.1 Eligibility criteria

3.1.1 Inclusion criteria

The following criteria for inclusion will be used to select studies to be included in the review:

i. Population of interest:
   - Adult population (16 years of age or older),
   - Adult sub-groups at increased risk of pneumococcal infection and often recommended for vaccination such as, amongst others, immunocompromised patients (e.g. HIV, cancer, functional or anatomic asplenia) and patients with COPD,
   - All countries.

ii. Intervention: marketed pneumococcal vaccines currently indicated for use in adults (PPV23 Pneumovax 23 (Merck/Sanofi Pasteur MSD) or PCV13 Prevenar 13 (Pfizer))

iii. Clinical outcomes of interest: pneumonia (pneumococcal and non-pneumococcal), other non-invasive disease, other diseases and clinical relevant outcomes. See section 3.5.1 for details.

iv. Effect measures of interest: estimates of vaccine effectiveness and impact in adults. See section 3.5.2 for details.

v. Setting: All (community, primary health care, outpatient, inpatient, hospital and emergency, secondary and tertiary health care, retirement/nursing homes)

vi. Study designs: Observational studies
   - Cohort, comparing risk or rates in vaccination and unvaccinated cohorts from the same population.
   - Case-control designs including conventional, indirect cohort design (Broome method), test-negative case-control, case-case comparison, and screening-method.
   - Surveillance-based assessments (e.g. temporal trends of reporting, laboratory confirmed cases)
   - Cluster randomized trials and step-wedge designs

vii. Timeframe: 1980 – 2015 October 15th (or date of last search run if later)

viii. Languages: English, French, Spanish, Portuguese, Dutch, German, Italian

3.1.2 Exclusion criteria

Studies with any of the following criteria will be excluded from the review:

i. No primary data. Review papers will be excluded. However, original studies included or discussed in the review, systematic or narrative, will be included.
ii. Studies restricted to children, i.e. with populations <16 years old.
iii. Randomized controlled trials, economic evaluation studies, transmission modeling studies.
iv. Studies focusing exclusively on invasive pneumococcal disease (defined as isolation of *Streptococcus pneumoniae* from a normally sterile body site (5)) and not including outcomes of interest as defined in section 3.5.1.
v. Studies on *S. pneumoniae* asymptomatic carriage
vi. Studies on immunogenicity.
vii. Non-human data (e.g. animal models, in-vitro, in-silico).

3.2 Information sources

3.2.1 Electronic databases and search strategy

We will conduct a literature search in MEDLINE (via PubMed), SCIELO and LILACS to obtain peer-reviewed, scientific publications of interest to the review. A detailed search strategy was developed for each bibliographic database (Annex 1).

The strategy that was chosen was option 2:

[Pubmed format]
(Pneumococcal Vaccines[MeSH] OR (Pneumococc*[tiab] AND (vaccin* or immuniz*[tiab] OR immunis*[tiab]))) OR (PPV23[tiab] OR "PPV 23"[tiab] OR Pneumovax[tiab] OR PCV13[tiab] OR "PCV 13"[tiab] OR Prevnar[tiab] OR Prevenar[tiab] OR "Pneumo 23"[tiab] OR Pneumo23[tiab]) OR (Vaccination[Mesh] AND pneumococcal infections[Mesh])
AND
(effectiveness[ti] OR effectiveness[ot] OR efficacy[ti] OR efficacy[ot] OR impact[ti] OR impact[ot] OR "treatment outcome"[mesh])

3.2.2 Grey literature selection

We will search for grey literature using a general search engine (e.g. Google) using target expressions, as well as target searches in public health institutions websites, namely the US CDC, Health Canada, Public health expertise and reference centre Québec, Public Health England, France InVS, Germany RKI, Australia Department of Health, Public Health Association of New Zealand, ECDC, WHO, Cochrane collaboration (Annex 2). Conference abstracts will not be included.

3.3 Data collection and analysis

3.3.1 Data management and storage

The results of the literature search will be imported into Endnote and MS Excel, where a track record of the selection process will be maintained with the reasons for exclusion/inclusion at each step (population, outcome, exposure, design).

3.3.2 Selection of studies

The selection process will take place in two steps. In the first step, two reviewers will independently review the titles and abstracts obtained from the electronic searches and apply the inclusion/exclusion criteria, differences will be resolved between the two reviewers. A third reviewer will resolve any remaining disagreements. The second selection step will be based on the full-text review of the articles retrieved in the first step. The flow of articles will be documented in a flowchart in the form of a PRISMA diagram in the report (6).

3.3.3 Reference checking and hand searching

Additionally, we will hand search the reference list of relevant studies retrieved from the electronic database search to identify additional studies.

3.3.4 Data collection

Data from the full-text selected papers will be extracted using a standard extraction form to ensure we gather all relevant data systematically and stored in MS Excel compatible format. A separate extraction table will be developed for each clinical outcome. The template will be piloted with 10 studies and modifications made if necessary. Data extraction will be carried out by a single reviewer; re-extraction of 10% of the papers will be done by a second reviewer.

3.4 Data items

The following information will be extracted:

- Reference ID / Study ID
- Reference (First Author last name, Journal, Year of publication)
- Country, region
- Vaccine type and brand(s)
- Study design
- Study population(s)
- Study period
- Setting(s)
- Age
- Clinical outcome(s) (as defined in section 3.5.1)
- Case finding and/or ascertainment method(s)
- Case definition
- Level of evidence of diagnosis certainty / case definition
- Clinical outcome severity
- Selection of controls (for case-control studies)
- Definition of control (for case-control studies)
- Definition of exposed (vaccinated)/ascertainment of exposure
- Selection unexposed (unvaccinated) (for cohort studies)Number of participants or person-time
- Number of (un)vaccinated subjects or (un)vaccinated person-time
- Number of (un)vaccinated subjects with clinical outcome
- Duration of follow up
- Effect measure(s) (as defined in section 3.5.2)
- Comorbidities adjusted for in the analysis (e.g. influenza infection, risk factors)
- Co-administration of vaccines (influenza, zoster)

- Serotypes (vaccine-type vs. non-vaccine-type) and method for serotype ascertainment (antigen test; brand and type)
- Uptake/coverage for pneumococcal vaccines and for influenza vaccines
- Study evidence grade
- Comments

One study may contribute more than one data point.

3.5 Outcomes

3.5.1 Clinical outcomes

- Pneumococcal pneumonia
    - Non-bacteremia pneumonia
    - All pneumococcal pneumonia combined (i.e. bacteremic and non-bacteremic pneumonia)
- All-cause pneumonia
- Other non-IPD disease
- Any-cause disease (including, but not limited to, e.g.: stroke, myocardial infarction, COPD exacerbation, asthma exacerbation, myocarditis, pericarditis, LRTI)
- Mortality or case fatality rate

The community-acquired pneumonia diagnosis categories used by Hak et al. in preparation for the CAPITA study can serve as a framework on the way different types of pneumonia will be classified (7).

3.5.2 Effect measures

The framework by Halloran et al. 2010 [8] will be used to define effectiveness and impact. Effectiveness has been split into direct effects (measured by comparing vaccinated and unvaccinated persons belonging to the same population and exposed to the same vaccination program) and indirect effects (population-level effects of widespread vaccination, as a result of reduced transmission; i.e. herd immunity); and the impact expresses the overall effect of the vaccination program on an entire population, including vaccinated and unvaccinated individuals(8, 9).

Example of effect measures for effectiveness are vaccine effectiveness (VE), relative risks (RR), odds ratios (OR), hazard ratio (HR). Examples of effect measures for impact are incidence rate ratios (IRR) and incidence rate differences.

Data will be extracted as reported in the publication and we will attempt to reclassify as per Halloran et al. 2010 [8] and Hanquet et al. 2013 [9].

3.6 Quality assessment

The GRADE framework and the Newcastle-Ottawa checklist for cohort and case-control studies, developed for assessing the quality of non-randomized studies in meta-analysis, will be used to assess the study limitations (risk of bias), and to inform the decision on whether to pursue meta-analysis (10-12). See Annex 3 for more details.

4 Analyses and reporting

The systematic review will be reported following the PRISMA statement (6). A flowchart will document the selection process. The differences between effectiveness and impact will be clearly defined.

The studies characteristics and effect measures will be summarized in tables. VE will be reported by study design and effect measure, for each clinical outcome and population, and the vaccine used will be specified. Impact will be reported by effect measure, for each clinical outcome, and the study period and the vaccine used will be specified.

A forest plot will display the estimates by type of vaccine used, study design and/or effect measure, and clinical outcome.

5 Meta-analysis

5.1 Decision criteria

Where data from more than one study on a given outcome is available, meta-analysis will be considered. The decision on whether or not to perform a meta-analysis of the results will depend on the factors listed below:
-Availability of effect measures that allow for meta-analysis
-Assessment of heterogeneity of study designs, effect measures, case definitions, study population
-Evidence of sufficient quality (see section 3.6)

5.2 Analysis

When the above requirements are fulfilled, meta-analysis will be performed using a random effects model to account for between-study heterogeneity. I^2 will be used to quantify the extent of heterogeneity. The results of the meta-analysis will be graphically displayed using forest plots. Meta-analyses will be performed using R and/or SAS 9.4.

Forest plots with summary estimates will be created for each vaccine type, by clinical outcome, and organized by study design/effect measure and population.

A detailed meta-analysis analysis plan will be developed following a decision to perform the meta-analysis.

6 References

1. Public Health England. Chapter 25: Pneumococcal London2013 [9 October 2015]. Available from: https://www.gov.uk/government/uploads/system/uploads/attachment_data/file/263318/Green-Book-Chapter-25-v5_2.pdf.
2. World Health Organization. Pneumococcal vaccines WHO position paper – 2012. Weekly epidemiological record. 2012;14(87):129-44.
3. Merck. PNEUMOVAX® 23 - Highlights of prescribing information 2015 [09 October 2015]. Available from:

https://www.merck.com/product/usa/pi_circulars/p/pneumovax_23/pneumovax_pi.pdf.
4. Pfizer. PREVNAR 13 - Highlights of prescribing information 2015 [09 October 2015]. Available from: http://labeling.pfizer.com/showlabeling.aspx?id=501.
5. CDC-NNDSS. Invasive Pneumococcal Disease (IPD) / Streptococcus pneumoniae, Invasive Disease (Streptococcus pneumoniae)
2010 Case Definition Atlanta, GA2015 [updated May 06, 2015; cited 2015 07 October 2015]. Available from: http://wwwn.cdc.gov/nndss/conditions/invasive-pneumococcal-disease/case-definition/2010/.
6. Moher D, Liberati A, Tetzlaff J, Altman DG. Preferred reporting items for systematic reviews and meta-analyses: the PRISMA statement. Annals of internal medicine. 2009;151(4):264-9.
7. Hak E, Grobbee D, Sanders E, Verheij T, Bolkenbaas M, Huijts S, et al. Rationale and design of CAPITA: a RCT of 13-valent conjugated pneumococcal vaccine efficacy among older adults. Neth J Med. 2008;66(9):378-83.
8. Halloran E, Longini IM, Struchiner CJ. Design and analysis of vaccine studies. New York: Springer; 2010.
9. Hanquet G, Valenciano M, Simondon F, Moren A. Vaccine effects and impact of vaccination programmes in post-licensure studies. Vaccine. 2013;31(48):5634-42.
10. Guyatt GH, Oxman AD, Vist GE, Kunz R, Falck-Ytter Y, Alonso-Coello P, et al. GRADE: an emerging consensus on rating quality of evidence and strength of recommendations. Bmj. 2008;336(7650):924-6.
11. Guyatt GH, Oxman AD, Vist G, Kunz R, Brozek J, Alonso-Coello P, et al. GRADE guidelines: 4. Rating the quality of evidence—study limitations (risk of bias). Journal of clinical epidemiology. 2011;64(4):407-15.
12. Wells GA, Shea B, O'Connell D, Peterson J, Welch V, Losos M, et al. The Newcastle-Ottawa Scale (NOS) for assessing the quality of nonrandomised studies in meta-analyses Ottawa: Otawa Hospital Research Institute; [cited 2015 8 October 2015]. Available from: http://www.ohri.ca/programs/clinical_epidemiology/oxford.asp.

Annex 1. Search strategy

Search strategy PubMed

Filters
Dates: 1 January 1980 to present
Languages: English, French, Spanish, Portuguese, Dutch, German, Italian

Search structure
Option 1 and option 2:
(Terms related to pneumococcal vaccines) AND (major outcomes)

Option 3:
(Terms related to pneumococcal vaccines) AND (major outcomes OR terms related to study design)

Objective: To identify all studies published in the literature with data on pneumococcal vaccine effectiveness and impact in adults.

Methodology: Literature search in PubMed

Search build (Number of PUBMED hits as of mid-October, 2015)

Search	Terms	Results	Comments
	**Vaccine**		
V#1	Pneumococcal Vaccines[MeSH]	5065	MeSH term introduced in 2001
V#2	Pneumococc*[tiab] AND (vaccin* or immuniz*[tiab] OR immunis*[tiab])	9097	Some articles might be missed by the MeSH term
V#3	("Streptococcus pneumoniae"[tiab] OR "S. pneumoniae"[tiab]) AND (vaccin* or immuniz*[tiab] OR immunis*[tiab])	4110	Theoretically the Streptococcus search terms are relevant, but doesn't look like yields relevant articles not found in #1 AND #2
V#4	Vaccination[Mesh] AND pneumococcal infections[Mesh]	1276	Mesh terms for vaccines and pneumococcal infections separately
V#5	PPV23[tiab] OR "PPV 23"[tiab] OR Pneumovax[tiab] OR PCV13[tiab] OR "PCV 13"[tiab] OR Prevnar[tiab] OR Prevenar[tiab] OR "Pneumo 23"[tiab] OR Pneumo23[tiab]	1028	Vaccines relevant for this review
V#6	#1 OR #2 OR #4 OR #5 (Pneumococcal Vaccines[MeSH] OR (Pneumococc*[tiab] AND (vaccin* or immuniz*[tiab] OR immunis*[tiab]))) OR (PPV23[tiab] OR "PPV 23"[tiab] OR Pneumovax[tiab] OR PCV13[tiab] OR "PCV 13"[tiab] OR Prevnar[tiab] OR Prevenar[tiab] OR "Pneumo 23"[tiab] OR Pneumo23[tiab]) OR (Vaccination[Mesh] AND pneumococcal infections[Mesh])	9973	
	**Vaccine AND outcome**		
	Searches with [tw]		

O#1	(effectiveness[tw] OR efficacy[tw] OR impact[tw])	1293686	
O#1b	(effectiveness[tw] OR efficacy[tw] OR impact[tw] OR effect*[ti] OR ineffect*[ti])		
O#2	V#6 AND (effectiveness[tw] OR efficacy[tw] OR impact[tw])	2390	Doesn't catch http://www.ncbi.nlm.nih.gov/pubmed/16773389 "Effect of prior pneumococcal vaccination on clinical outcome of hospitalized adults with community-acquired pneumococcal pneumonia."
O#2a	V#6 AND (effectiveness[tw] OR efficacy[tw] OR impact[tw]) NOT (Randomized Controlled Trial[ptyp])	2248	
O#3	V#6 AND (effectiveness[tw] OR efficacy[tw] OR impact[tw] OR "treatment outcome"[mesh])	2628	"treatment outcome"[mesh] does catch the article above
O#3b	V#6 AND (effectiveness[tw] OR efficacy[tw] OR impact[tw] OR effect*[ti] OR ineffect*[ti] OR "treatment outcome"[mesh])	2637	
O#3a	V#6 AND (effectiveness[tw] OR efficacy[tw] OR impact[tw] OR "treatment outcome"[mesh]) NOT (Randomized Controlled Trial[ptyp])	2448	
	Searches with [ti] and [ot] (title and author-supplied keywords, if available)		
O#4	(effectiveness[ti] OR effectiveness[ot] OR efficacy[ti] OR efficacy[ot] OR impact[ti] OR impact[ot])	285054	
O#5	V#6 AND (effectiveness[ti] OR effectiveness[ot] OR efficacy[ti] OR efficacy[ot] OR impact[ti] OR impact[ot])	776	
O#5a	V#6 AND (effectiveness[ti] OR effectiveness[ot] OR efficacy[ti] OR efficacy[ot] OR impact[ti] OR impact[ot]) NOT (Randomized Controlled Trial[ptyp])	719	
O#6	V#6 AND "treatment outcome"[mesh]	396	Treatment outcome[mesh]: Evaluation undertaken to assess the results or consequences of management and procedures used in combating disease in order to determine the efficacy, effectiveness, safety, and practicability of these interventions in individual cases or series.
O#7	(effectiveness[ti] OR effectiveness[ot] OR efficacy[ti] OR efficacy[ot] OR impact[ti] OR impact[ot] OR "treatment outcome"[mesh])	913517	
O#7b	(effect*[ti] OR effect*[ot] OR efficacy[ti] OR efficacy[ot] OR impact[ti] OR impact[ot] OR ineffect*[ti] OR "treatment outcome"[mesh])	1981198	Changed effectiveness to effect* and added ineffect*[ti]
O#8	V#6 AND (effectiveness[ti] OR effectiveness[ot] OR efficacy[ti] OR efficacy[ot] OR impact[ti] OR impact[ot] OR "treatment outcome"[mesh])	1091	
O#8a	V#6 AND (effectiveness[ti] OR effectiveness[ot] OR efficacy[ti] OR efficacy[ot] OR impact[ti] OR impact[ot] OR "treatment outcome"[mesh]) NOT (Randomized Controlled Trial[ptyp])	993	NOT RCT

O#8b	V#6 AND (effect*[ti] OR effect*[ot] OR efficacy[ti] OR efficacy[ot] OR impact[ti] OR impact[ot] OR ineffect*[ti] OR "treatment outcome"[mesh])	1453	Changed effectiveness to effect* and added ineffect*[ti]
	**Vaccine AND (outcome OR study design)**		
S#1	(Observational[tw] OR case-control[tw] OR cohort[tw] OR surveillance [tw])	782092	Major study types
S#2	(Observational[tw] OR case-control[tw] OR cohort[tw])	650564	Major study types except surveillance
S#3	V#6AND (O#1 OR #S1)	3637	Outcome with [tw]; study design with surveillance[tw]
S#3a	V#6AND (O#1 OR #S1) NOT Randomized Controlled Trial[ptyp]	3466	
S#4	V#6 AND (O#1 OR #S2)	2946	Outcome with [tw]; study design without surveillance[tw]
S#4a	V#6 AND (O#1 OR #S2) NOT Randomized Controlled Trial[ptyp]	2780	
S#5	V#6 AND (O#4 OR #S1)	2439	Outcome with [ti] and [ot]; study design with surveillance[tw]
S#5a	V#6 AND (O#4 OR #S1) NOT Randomized Controlled Trial[ptyp]	2337	
S#6	V#6 AND (O#4 OR #S2)	1520	Outcome with [ti] and [ot]; study design without surveillance[tw]
S#6a	V#6 AND (O#4 OR #S2) NOT Randomized Controlled Trial[ptyp]	1427	
S#7	V#6 AND (O#7 OR #S1)	2686	Outcome with [ti] and [ot] and "treatment outcome"[mesh]; study design with surveillance[tw]
S#7a	V#6 AND (O#7 OR #S1) NOT Randomized Controlled Trial[ptyp]	2544	
S#8	V#6 AND (O#7 OR #S2)	1793	Outcome with [ti] and [ot] and "treatment outcome"[mesh]; study design without surveillance[tw]
S#8a	V#6 AND (O#7 OR #S2) NOT Randomized Controlled Trial[ptyp]	1659	
S#8b	V#6 AND (O#7b OR #S2)	2103	Effect* instead of effect*

Search PUBMED (nr of hits on Oct 30, 2015)

Filters Dates: 1 Jan 1980 to present
Languages: English, French, Spanish, Portuguese, Dutch, German, Italian

Option 1: *#O3, 2637 hits* **Vaccine AND outcome**
Option 1a: *#O3b, 2896 hits*
Option 2: *#O8, 1092 hits* **Vaccine AND outcome**
Option 2a: *#O8b, 1454 hits*
Option 3: *#S8, 1796 hits* **Vaccine AND (outcome OR study design)**
Option 3a: *#S8b, 2105 hits*

The option that was chosen is option 2:

(Pneumococcal Vaccines[MeSH] OR (Pneumococc*[tiab] AND (vaccin* or immuniz*[tiab] OR immunis*[tiab]))) OR (PPV23[tiab] OR "PPV 23"[tiab] OR Pneumovax[tiab] OR PCV13[tiab] OR "PCV 13"[tiab] OR Prevnar[tiab] OR Prevenar[tiab] OR "Pneumo 23"[tiab] OR Pneumo23[tiab]) OR (Vaccination[Mesh] AND pneumococcal infections[Mesh])
AND
(effectiveness[ti] OR effectiveness[ot] OR efficacy[ti] OR efficacy[ot] OR impact[ti] OR impact[ot] OR "treatment outcome"[mesh])

Search strategy SCIELO

Filters Regional- broadest scope
All indexes

Search structures
Vaccine AND outcome

Search	Terms	Results	Comments
	**Vaccine**		
#1	(Pneumococcal AND vaccin*) OR (vacuna* AND (neumococo OR Neumocóccica OR neumocócica)) OR (vacina* AND pneumocócica) OR prevnar OR prevenar OR (pneumo 23)	221	Vaccine terms in English, Spanish, Portuguese
	(Pneumococcal AND vaccin*)	191	
	(vacuna* AND (neumococo OR Neumocóccica OR neumocócica))	109	
	(vacina* AND pneumocócica)	16	
	prevnar	3	
	prevenar	6	
	Pneumovax	0	
	Pneumo 23	4	
	**Outcome**		
#2	Effectiveness OR efectividad OR efetividade OR efficacy OR eficacia OR impact OR impacto	38404	Outcome terms in English, Spanish, Portuguese
	effectiveness	8409	
	efectividad	3563	
	efetividade	1659	
	efficacy	6713	
	eficacia	9113	
	eficácia	9113	
	impact	20405	
	impacto	15422	
	**Vaccine AND outcome**		
#3	#1 AND #2	70	Vaccine AND outcome

Search SCIELO
((Pneumococcal AND vaccin*) OR (vacuna* AND neumococo) OR (vacina* AND pneumocócica) OR prevnar OR prevenar OR pneumo 23) AND (Effectiveness OR efectividad OR efetividade OR efficacy OR eficacia OR impact OR impacto)

Filters Regional (by default; and it is the broadest one)
All indexes

Search strategy LILACS

Search	Terms	Results	Comments
	**Vaccine**		
#1	(Pneumococcal AND (vaccine OR vaccination)) OR (vacuna AND (neumococo OR Neumocóccica OR neumocócica)) OR (vacina AND pneumocócica) OR prevnar OR prevenar OR pneumovax OR pneumo23	215	Vaccine terms in English, Spanish, Portuguese
	(Pneumococcal AND (vaccine OR vaccination))	178	
	(vacuna AND (neumococo OR Neumocóccica OR neumocócica))	123	
	(vacina AND pneumocócica)	57	
	prevnar	2	
	prevenar	3	
	Pneumovax	2	
	Pneumo 23	0	
	Pneumo23	2	
	**Outcome**		
#2	Effectiveness OR efectividad OR efetividade OR efficacy OR eficacia OR impact OR impacto	47823	Outcome terms in English, Spanish, Portuguese
	effectiveness	13929	
	efectividad	5148	
	efetividade	10905	
	efficacy	6411	
	eficacia	21006	
	eficácia	21006	
	impact	15717	
	impacto	13528	
	**Vaccine AND outcome**		
#3	#1 AND #2	58	Vaccine AND outcome

Search LILACS

((Pneumococcal AND (vaccine OR vaccination)) OR (vacuna AND (neumococo OR Neumocóccica OR neumocócica)) OR (vacina AND pneumocócica) OR prevnar OR prevenar OR pneumovax OR pneumo23) AND (Effectiveness OR efectividad OR efetividade OR efficacy OR eficacia OR impact OR impacto)

Annex 2. Grey literature search

Search terms to be combined in Google:

- Pneumococcal vaccine effectiveness,
- *Streptococcus pneumonia* vaccine effectiveness
- Adults
- Surveillance
- Before after introduction
- Trends

The first 100 hits will be examined.

Websites URLs:

- Australia department of health – Immunise Australia http://www.immunise.health.gov.au/
- Cochrane Collaboration http://www.cochrane.org/
- European Centre for Disease Prevention and Control http://ecdc.europa.eu/
- French Institute for Public Health Surveillance http://www.invs.sante.fr/en
- Germany Robert Koch Institute (RKI) http://www.rki.de/
- Health Canada http://www.hc-sc.gc.ca/
- Public Health Association of New Zealand http://www.pha.org.nz/
- Public Health England https://www.gov.uk/government/organisations/public-health-england
- Public Health Expertise and reference centre Québec https://www.inspq.qc.ca/en
- US CDChttp://www.cdc.gov/
- World Health Organization http://www.who.int/

Annex 3. Risk of bias tools

GRADE

Study limitations in observational studies (11)

1. Failure to develop and apply appropriate eligibility criteria (inclusion of control population)
   - Under- or overmatching in case-control studies
   - Selection of exposed and unexposed in cohort studies from different populations
2. Flawed measurement of both exposure and outcome
   - Differences in measurement of exposure (e.g. recall bias in case-control studies)
   - Differential surveillance for outcome in exposed and unexposed in cohort studies
3. Failure to adequately control confounding
   - Failure to accurate measurement of all known prognostic factors
   - Failure to match for prognostic factors and/or lack of adjustment in statistical analysis
4. Incomplete follow-up

<u>Factors decreasing confidence</u>
Limitation in study design
Inconsistency
Indirectness
Imprecision
Publication bias

<u>Factors increasing confidence</u>
Large effect
Dose-response
Antagonistic bias

Newcastle Ottawa scale for case-control and cohort studies (12)

NEWCASTLE -OTTAWA QUALITY ASSESSMENT SCALE
COHORT STUDIES

Note: A study can be awarded a maximum of one star for each numbered item within the Selection and Outcome categories. A maximum of two stars can be given for Comparability

Selection

1) Representativeness of the exposed cohort
   a) truly representative of the average ____________ (describe) in the community *
   b) somewhat representative of the average ____________ in the community *
   c) selected group of users e.g. nurses, volunteers
   d) no description of the derivation of the cohort

2) Selection of the non exposed cohort

a) drawn from the same community as the exposed cohort *
b) drawn from a different source
c) no description of the derivation of the non exposed cohort

3) Ascertainment of exposure
a) secure record (e.g. surgical records) *
b) structured interview *
c) written self report
d) no description

4) Demonstration that outcome of interest was not present at start of study
a) yes *
b) no

Comparability

1) Comparability of cohorts on the basis of the design or analysis
a) study controls for ____________ (select the most important factor) *
b) study controls for any additional factor * (This criteria could be modified to indicate specific control for a second important factor.)

Outcome

1) Assessment of outcome
a) independent blind assessment *
b) record linkage *
c) self report
d) no description

2) Was follow-up long enough for outcomes to occur
a) yes (select an adequate follow up period for outcome of interest) *
b) no

3) Adequacy of follow up of cohorts
a) complete follow up -all subjects accounted for *
b) subjects lost to follow up unlikely to introduce bias -small number lost -> ____ % (select an adequate %)follow up, or description provided of those lost) *
c) follow up rate < ____% (select an adequate %) and no description of those lost
d) no statement

NEWCASTLE -OTTAWA QUALITY ASSESSMENT SCALE
CASE CONTROL STUDIES

Note: A study can be awarded a maximum of one star for each numbered item within the Selection and Exposure categories. A maximum of two stars can be given for Comparability.

Selection

1) Is the case definition adequate?
a) yes, with independent validation *
b) yes, e.g. record linkage or based on self reports
c) no description

2) Representativeness of the cases
a) consecutive or obviously representative series of cases *
b) potential for selection biases or not stated

3) Selection of Controls

a) community controls *
b) hospital controls
c) no description

4) Definition of Controls
a) no history of disease (endpoint) *
b) no description of source

Comparability

1) Comparability of cases and controls on the basis of the design or analysis
a) study controls for _______________ (Select the most important factor.) *
b) study controls for any additional factor * (This criteria could be modified to indicate specific control for a second important factor.)

Exposure

1)Ascertainment of exposure
a) secure record (e.g. surgical records) *
b) structured interview where blind to case/control status *
c) interview not blinded to case/control status
d) written self report or medical record only
e) no description

2) Same method of ascertainment for cases and controls
a) yes *
b) no

3) Non-Response rate
a) same rate for both groups *
b) non respondents described
c) rate different and no designation
