# Supplementary material for: Effectiveness of pneumococcal vaccines in preventing pneumonia in adults, a systematic review and meta-analyses of observational studies
Source: PLoS One. 2017 May 23;12(5):e0177985. doi: 10.1371/journal.pone.0177985 (PMC5441633; doi:10.1371/journal.pone.0177985)
Supplement: S4 File — (DOCX) [file pone.0177985.s004.docx]

**References for all included studies**

Ansaldi F, Turello V, Lai P, Bastone G, De Luca S, Rosselli R, et al. Effectiveness of a 23-valent polysaccharide vaccine in preventing pneumonia and non-invasive pneumococcal infection in elderly people: a large-scale retrospective cohort study. J Int Med Res. 2005;33(5):490-500. Epub 2005/10/15. PubMed PMID: 16222881.

Chiou WY, Hung SK, Lai CL, Lin HY, Su YC, Chen YC, et al. Effect of 23-Valent Pneumococcal Polysaccharide Vaccine Inoculated During Anti-Cancer Treatment Period in Elderly Lung Cancer Patients on Community-Acquired Pneumonia Hospitalization: A Nationwide Population-Based Cohort Study. Medicine (Baltimore). 2015;94(26):e1022. Epub 2015/07/02. doi: 10.1097/md.0000000000001022. PubMed PMID: 26131806; PubMed Central PMCID: PMCPmc4504648.

Christenson B, Hedlund J, Lundbergh P, Ortqvist A. Additive preventive effect of influenza and pneumococcal vaccines in elderly persons. Eur Respir J. 2004;23(3):363-8. Epub 2004/04/07. PubMed PMID: 15065822.

Curran A, Falco V, Crespo M, Martinez X, Ribera E, Villar del Saz S, et al. Bacterial pneumonia in HIV-infected patients: use of the pneumonia severity index and impact of current management on incidence, aetiology and outcome. HIV Med. 2008;9(8):609-15. Epub 2008/06/19. doi: 10.1111/j.1468-1293.2008.00603.x. PubMed PMID: 18557951.

Dominguez A, Izquierdo C, Salleras L, Ruiz L, Sousa D, Bayas JM, et al. Effectiveness of the pneumococcal polysaccharide vaccine in preventing pneumonia in the elderly. Eur Respir J. 2010;36(3):608-14. Epub 2010/01/16. doi: 10.1183/09031936.00171309. PubMed PMID: 20075048.

Eurich DT, Johnstone JJ, Minhas-Sandhu JK, Marrie TJ, Majumdar SR. Pneumococcal vaccination and risk of acute coronary syndromes in patients with pneumonia: population-based cohort study. Heart. 2012;98(14):1072-7. Epub 2012/06/29. doi: 10.1136/heartjnl-2012-301743. PubMed PMID: 22739637.

Gable CB, Holzer SS, Engelhart L, Friedman RB, Smeltz F, Schroeder D, et al. Pneumococcal vaccine. Efficacy and associated cost savings. JAMA. 1990;264(22):2910-5. Epub 1990/12/12. PubMed PMID: 2232086.

Guerrero M, Kruger S, Saitoh A, Sorvillo F, Cheng KJ, French C, et al. Pneumonia in HIV-infected patients: a case-control survey of factors involved in risk and prevention. Aids. 1999;13(14):1971-5. Epub 1999/10/08. PubMed PMID: 10513657.

Hechter RC, Chao C, Jacobsen SJ, Slezak JM, Quinn VP, Van Den Eeden SK, et al. Clinical effectiveness of pneumococcal polysaccharide vaccine in men: California Men's Health Study. Vaccine. 2012;30(38):5625-30. Epub 2012/07/14. doi: 10.1016/j.vaccine.2012.06.085. PubMed PMID: 22789510.

Hung CC, Chen MY, Hsieh SM, Hsiao CF, Sheng WH, Chang SC. Clinical experience of the 23-valent capsular polysaccharide pneumococcal vaccination in HIV-1-infected patients receiving highly active antiretroviral therapy: a prospective observational study. Vaccine. 2004;22(15-16):2006-12. Epub 2004/05/04. doi: 10.1016/j.vaccine.2003.10.030. PubMed PMID: 15121313.

Hung IF, Leung AY, Chu DW, Leung D, Cheung T, Chan CK, et al. Prevention of acute myocardial infarction and stroke among elderly persons by dual pneumococcal and influenza vaccination: a prospective cohort study. Clin Infect Dis. 2010;51(9):1007-16. Epub 2010/10/05. doi: 10.1086/656587. PubMed PMID: 20887208.

Jackson LA, Neuzil KM, Yu O, Benson P, Barlow WE, Adams AL, et al. Effectiveness of pneumococcal polysaccharide vaccine in older adults. N Engl J Med. 2003;348(18):1747-55. Epub 2003/05/02. doi: 10.1056/NEJMoa022678. PubMed PMID: 12724480.

Johnstone J, Eurich DT, Minhas JK, Marrie TJ, Majumdar SR. Impact of the pneumococcal vaccine on long-term morbidity and mortality of adults at high risk for pneumonia. Clin Infect Dis. 2010;51(1):15-22. Epub 2010/05/28. doi: 10.1086/653114. PubMed PMID: 20504233.

Leventer-Roberts M, Feldman BS, Brufman I, Cohen-Stavi CJ, Hoshen M, Balicer RD. Effectiveness of 23-valent pneumococcal polysaccharide vaccine against invasive disease and hospital-treated pneumonia among people aged >/=65 years: a retrospective case-control study. Clin Infect Dis. 2015;60(10):1472-80. Epub 2015/02/12. doi: 10.1093/cid/civ096. PubMed PMID: 25669354.

Lindenburg CE, Langendam MW, Benthem BHv, Miedema F, Coutinho RA. No evidence that vaccination with a polysaccharide pneumococcal vaccine protects drug users against all-cause pneumonia. Aids. 2001;15(10):1315-7.

Lopez-Palomo C, Martin-Zamorano M, Benitez E, Fernandez-Gutierrez C, Guerrero F, Rodriguez-Iglesias M, et al. Pneumonia in HIV-infected patients in the HAART era: incidence, risk, and impact of the pneumococcal vaccination. J Med Virol. 2004;72(4):517-24. Epub 2004/02/26. doi: 10.1002/jmv.20045. PubMed PMID: 14981752.

Musher DM, Rueda-Jaimes AM, Graviss EA, Rodriguez-Barradas MC. Effect of pneumococcal vaccination: a comparison of vaccination rates in patients with bacteremic and nonbacteremic pneumococcal pneumonia. Clin Infect Dis. 2006;43(8):1004-8. Epub 2006/09/20. doi: 10.1086/507699. PubMed PMID: 16983612.

Navin TR, Rimland D, Lennox JL, Jernigan J, Cetron M, Hightower A, et al. Risk factors for community-acquired pneumonia among persons infected with human immunodeficiency virus. J Inf Dis. 2000;181(1):158-64.

Nichol KL. The additive benefits of influenza and pneumococcal vaccinations during influenza seasons among elderly persons with chronic lung disease. Vaccine. 1999;17:S91-S3.

Nichol KL, Baken L, Wuorenma J, Nelson A. The health and economic benefits associated with pneumococcal vaccination of elderly persons with chronic lung disease. Arch Intern Med. 1999;159(20):2437-42. Epub 2000/02/09. PubMed PMID: 10665892.

Ochoa-Gondar O, Vila-Corcoles A, Ansa X, Rodriguez-Blanco T, Salsench E, de Diego C, et al. Effectiveness of pneumococcal vaccination in older adults with chronic respiratory diseases: results of the EVAN-65 study. Vaccine. 2008;26(16):1955-62. Epub 2008/03/18. doi: 10.1016/j.vaccine.2008.02.021. PubMed PMID: 18343541.

Ochoa-Gondar O, Vila-Corcoles A, Rodriguez-Blanco T, Gomez-Bertomeu F, Figuerola-Massana E, Raga-Luria X, et al. Effectiveness of the 23-valent pneumococcal polysaccharide vaccine against community-acquired pneumonia in the general population aged >/= 60 years: 3 years of follow-up in the CAPAMIS study. Clin Infect Dis. 2014;58(7):909-17. Epub 2014/02/18. doi: 10.1093/cid/ciu002. PubMed PMID: 24532544.

Rodriguez-Barradas MC, Goulet J, Brown S, Goetz MB, Rimland D, Simberkoff MS, et al. Impact of pneumococcal vaccination on the incidence of pneumonia by HIV infection status among patients enrolled in the Veterans Aging Cohort 5-Site Study. Clin Infect Dis. 2008;46(7):1093-100. Epub 2008/05/01. doi: 10.1086/529201. PubMed PMID: 18444830; PubMed Central PMCID: PMCPmc3115628.

Skull SA, Andrews RM, Byrnes GB, Kelly HA, Nolan TM, Brown GV, et al. Prevention of community-acquired pneumonia among a cohort of hospitalized elderly: benefit due to influenza and pneumococcal vaccination not demonstrated. Vaccine. 2007;25(23):4631-40. Epub 2007/05/05. doi: 10.1016/j.vaccine.2007.03.015. PubMed PMID: 17478015.

Song JY, Lee JS, Wie SH, Kim HY, Lee J, Seo YB, et al. Prospective cohort study on the effectiveness of influenza and pneumococcal vaccines in preventing pneumonia development and hospitalization. Clin Vaccine Immunol. 2015;22(2):229-34. Epub 2014/12/30. doi: 10.1128/cvi.00673-14. PubMed PMID: 25540271; PubMed Central PMCID: PMCPmc4308868.

Teshale EH, Hanson D, Flannery B, Phares C, Wolfe M, Schuchat A, et al. Effectiveness of 23-valent polysaccharide pneumococcal vaccine on pneumonia in HIV-infected adults in the United States, 1998--2003. Vaccine. 2008;26(46):5830-4. Epub 2008/09/13. doi: 10.1016/j.vaccine.2008.08.032. PubMed PMID: 18786586.

Tsai YH, Hsieh MJ, Chang CJ, Wen YW, Hu HC, Chao YN, et al. The 23-valent pneumococcal polysaccharide vaccine is effective in elderly adults over 75 years old--Taiwan's PPV vaccination program. Vaccine. 2015;33(25):2897-902. Epub 2015/05/06. doi: 10.1016/j.vaccine.2015.04.068. PubMed PMID: 25936662.

Vila-Corcoles A, Ochoa Gondar, O., Marín Canseco, M. L., Guinea Oruechevarría, I., Álvarez Luy, M. . Efectividad de la vacuna antineumocócica en pacientes mayores de 65 años. Medifam. 2003;13(4):61-8.

Vila-Corcoles A, Ochoa-Gondar O, Hospital I, Ansa X, Vilanova A, Rodriguez T, et al. Protective effects of the 23-valent pneumococcal polysaccharide vaccine in the elderly population: the EVAN-65 study. Clin Infect Dis. 2006;43(7):860-8. Epub 2006/08/31. doi: 10.1086/507340. PubMed PMID: 16941367.

Vila-Corcoles A, Salsench E, Rodriguez-Blanco T, Ochoa-Gondar O, de Diego C, Valdivieso A, et al. Clinical effectiveness of 23-valent pneumococcal polysaccharide vaccine against pneumonia in middle-aged and older adults: a matched case-control study. Vaccine. 2009;27(10):1504-10. Epub 2009/01/28. doi: 10.1016/j.vaccine.2009.01.013. PubMed PMID: 19171174.

Vila-Corcoles A, Ochoa-Gondar O, Rodriguez-Blanco T, Gutierrez-Perez A, Vila-Rovira A. Clinical effectiveness of 23-valent pneumococcal polysaccharide vaccine against pneumonia in patients with chronic pulmonary diseases: a matched case-control study. Hum Vaccin Immunother. 2012;8(5):639-44. Epub 2012/05/29. doi: 10.4161/hv.19466. PubMed PMID: 22634442.

Wagner C, Popp W, Posch M, Vlasich C, Rosenberger-Spitzy A. Impact of pneumococcal vaccination on morbidity and mortality of geriatric patients: a case-controlled study. Gerontology. 2003;49(4):246-50. Epub 2003/06/07. doi: 70405. PubMed PMID: 12792160.

Wiemken TL, Carrico RM, Klein SL, Jonsson CB, Peyrani P, Kelley RR, et al. The effectiveness of the polysaccharide pneumococcal vaccine for the prevention of hospitalizations due to Streptococcus pneumoniae community-acquired pneumonia in the elderly differs between the sexes: results from the Community-Acquired Pneumonia Organization (CAPO) international cohort study. Vaccine. 2014;32(19):2198-203. Epub 2014/03/13. doi: 10.1016/j.vaccine.2014.02.048. PubMed PMID: 24613522.
