# Supplementary figures and images for: Effectiveness of pneumococcal vaccines in preventing pneumonia in adults, a systematic review and meta-analyses of observational studies
Source: PLoS One. 2017 May 23;12(5):e0177985. doi: 10.1371/journal.pone.0177985 (PMC5441633; doi:10.1371/journal.pone.0177985)

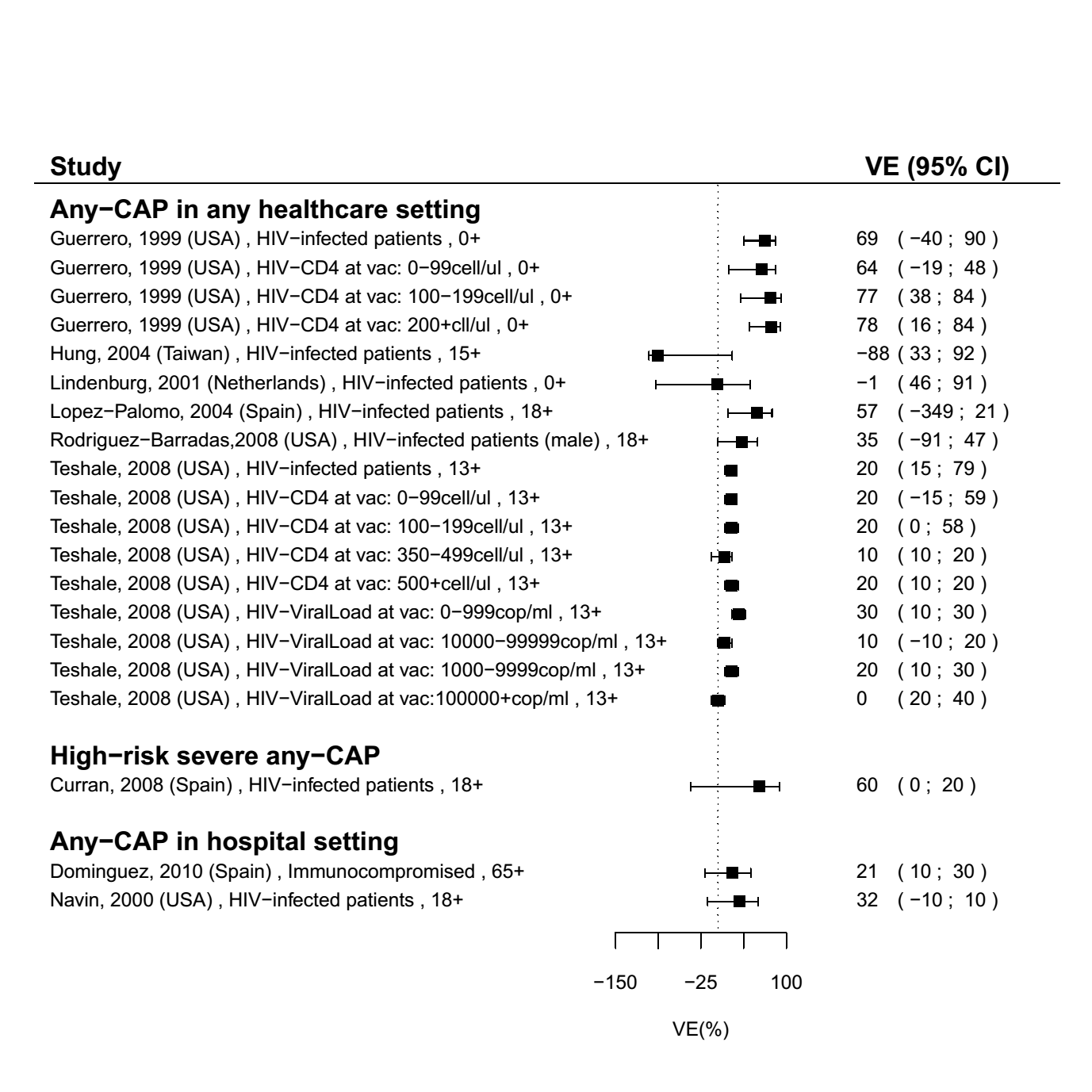

Supplement: S1 Fig — (TIF) [file pone.0177985.s005.tif]

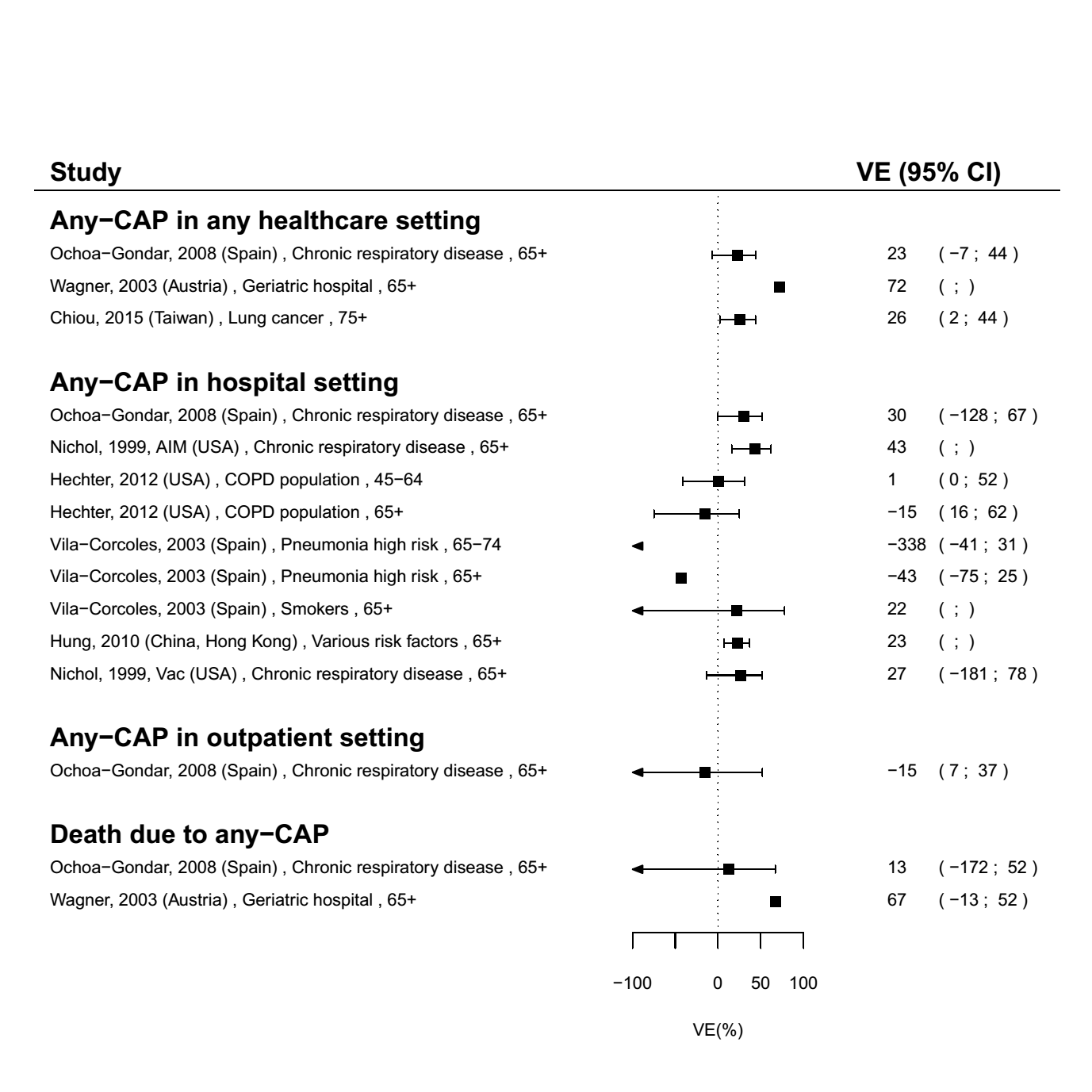

Supplement: S2 Fig — (TIF) [file pone.0177985.s006.tif]
