# Supplementary material for: Effectiveness of pneumococcal vaccines in preventing pneumonia in adults, a systematic review and meta-analyses of observational studies
Source: PLoS One. 2017 May 23;12(5):e0177985. doi: 10.1371/journal.pone.0177985 (PMC5441633; doi:10.1371/journal.pone.0177985)
Supplement: S1 Table — (DOCX) [file pone.0177985.s007.docx]

S1 Table. Quality assessment of included studies using the Newcastle-Ottawa Score [13]

| Reference | Study design | Selection (/4) | Comparability (/2) | Exposure or Outcome (/3) | Total (/9) |
| --- | --- | --- | --- | --- | --- |
| Ansaldi, J Int Med Res, 2005 | Self-controlled-risk-windows | 3 | 2 | 2 | 7 |
| Chiou, 2015, Medicine | Cohort | 3 | 2 | 2 | 7 |
| Christenson, 2004, ERJ | Cohort | 3 | 1 | 2 | 6 |
| Curran, 2008, HIV Med | Case-control | 3 | 0 | 2 | 5 |
| Dominguez, 2010, ERJ | Case-control | 2 | 2 | 3 | 7 |
| Eurich, 2012 | Cohort | 3 | 1 | 2 | 6 |
| Gable, 1990, JAMA | Cohort | 2 | 0 | 3 | 5 |
| Guerrero, 1999, AIDS | Case-control | 3 | 2 | 1 | 6 |
| Hechter, 2012, Vaccine | Cohort | 2 | 2 | 2 | 6 |
| Hung, 2004, Vaccine | Cohort | 2 | 1 | 1 | 4 |
| Hung, 2010 | Cohort | 3 | 0 | 3 | 6 |
| Jackson, 2003, NEJM | Cohort | 2 | 2 | 2 | 6 |
| Johnstone, 2010, Clin Infect Dis | Cohort | 3 | 2 | 2 | 7 |
| Leventer-Roberts, 2015, Clin Inf Dis | Case-control | 3 | 2 | 3 | 8 |
| Lindenburg, 2001, AIDS | Cohort | 2 | 0 | 2 | 4 |
| Lopez-Palomo, 2004, J Med Virology | Cohort | 3 | 0 | 2 | 5 |
| Musher, 2006, Clin Inf Dis | Case-control | 2 | 0 | 3 | 5 |
| Navin, 2000, JID | Case-control | 2 | 1 | 1 | 4 |
| Nichol, 1999, Arch Intern Med | Cohort | 2 | 2 | 2 | 6 |
| Nichol, 1999, Vaccine | Cohort | 2 | 2 | 1 | 5 |
| Ochoa-Gondar, 2008, Vaccine: EVAN 65 | Cohort | 3 | 2 | 2 | 7 |
| Ochoa-Gondar, Clinical Infectious Diseases, 2014: CAPAMIS | Cohort | 3 | 2 | 2 | 7 |
| Rodriguez-Barradas_2008_ClinInfDis | Cohort | 2 | 1 | 2 | 5 |
| Skull, 2007, Vaccine | Case-cohort | 1 | 2 | 3 | 6 |
| Song, Clin Vacc Immunol, 2015 | Case-control | 2 | 1 | 1 | 4 |
| Teshale, 2008, Vaccine | Cohort | 2 | 2 | 2 | 6 |
| Tsai, 2015, Vaccine | Cohort | 3 | 2 | 2 | 7 |
| Vila-Corcoles, 2003, Medifam | Cohort | 2 | 0 | 1 | 3 |
| Vila-Corcoles, 2006, CID; EVAN 65 | Cohort | 3 | 2 | 2 | 7 |
| Vila-Corcoles, 2009, Vaccine | Case-control | 4 | 1 | 3 | 8 |
| Vila-Corcoles, 2012, Hum Vacc Immuno | Case-control | 3 | 1 | 3 | 7 |
| Wagner, 2003, Gerontology | Case-control | 1 | 1 | 1 | 3 |
| Wiemken, 2014, Vaccine | Case-control | 3 | 2 | 2 | 7 |
