# Supplementary material for: Effectiveness of pneumococcal vaccines in preventing pneumonia in adults, a systematic review and meta-analyses of observational studies
Source: PLoS One. 2017 May 23;12(5):e0177985. doi: 10.1371/journal.pone.0177985 (PMC5441633; doi:10.1371/journal.pone.0177985)
Supplement: S2 Table — (DOCX) [file pone.0177985.s008.docx]

S2 Table. Study characteristics and results for any-CAP

| Author, year of publication (country) | Population | Population source | Study design | Study period | Healthcare setting | Age (years) | Vaccinated CAP cases | VE(%) | 95%CI |
| --- | --- | --- | --- | --- | --- | --- | --- | --- | --- |
| *General population* |  |  |  |  |  |  |  |  |  |
| Song, 2015 (Republic of Korea) | General population | ILI patients at emergency rooms | Case-control (P) | 2013-2014 | Any setting or severity | 19+ | 14 | -48 | -220; 32 |
|  |  |  |  |  |  | 19-64 | 2 | -143 | -1501; 63 |
|  |  |  |  |  |  | 65+ | 12 | -34 | -223; 44 |
| Vila-Corcoles, 2006 (Spain) | General population | Primary care centers | Cohort (P) | 2002-2005 | Any setting or severity | 65+ | 266 | 21 | 2; 36 |
| Gable, 1990 (United States) | General population | Claims data | Cohort (R) | 1986-1988 | Any setting or severity | 51+ | NA | 69 | 48; 81 |
| Vila-Corcoles, 2006 (Spain) | General population | Primary care centers | Cohort (P) | 2002-2005 | Death | 65+ | 26 | 59 | 28; 77 |
| Vila-Corcoles, 2003 (Spain) | General population | Primary care centers | Cohort (R) | 1994-1999 | Death | 65+ | NA | 13 | NA |
| Tsai, 2015 (Taiwan) | General population | Health insurance database | Cohort (R) | 2009 | Death | 75+ | 153 | 93 | 92; 94 |
| Christenson, 2004 (Sweden) | General population | Stockholm County | Cohort (P) | 1999-2000 | Death | 65+ | NA | 7 | -19; 28 |
| Ochoa-Gondar, 2014 (Spain) | General population | Primary care centers | Cohort (P) | 2008-2011 | Death | 60+ | 27 | -4 | -69; 36 |
| Leventer-Roberts, 2015 (Israel) | General population | Health maintenance organization | Case-control ® | 2008-2010 | Hospitalization | 65+ | NA | -1 | -4; 3 |
|  |  |  |  |  |  | 65-74 | NA | -12 | -21; -3 |
|  |  |  |  |  |  | 75+ | NA | 3 | -1; 8 |
| Hechter, 2012 (United States) | General population | Health maintenance organization | Cohort (P) | 2002-2009 | Hospitalization | 45-64 | 250 | -18 | -37; -2 |
|  |  |  |  |  |  | 65+ | 158 | 5 | -17; 22 |
| Johnstone, 2010 (Canada) | General population | CAP patients discharged from hospital | Cohort (P) (repeat CAP admission) | 2000-2007 | Hospitalization | 18+ | 164 | -1 | -30; 21 |
| Dominguez, 2010 (Spain) | General population | Hospital | Case-control (P&R) | 2005-2007 | Hospitalization | 65+ | 229 | 24 | 1; 41 |
| Vila-Corcoles, 2006 (Spain) | General population | Primary care centers | Cohort (P) | 2002-2005 | Hospitalization | 65+ | 194 | 26 | 8; 41 |
| Ansaldi, 2005 (Italy) | General population | Vaccination centers and nursing homes | Self-controlled-risk-windows (R) | 1998-2002 | Hospitalization | 64+ | 123 | 28 | 7; 43 |
| Jackson, 2003 (United States) | General population | Health maintenance organization | Cohort (R) | 1998-2001 | Hospitalization | 65+ | NA | -14 | -28; -2 |
| Vila-Corcoles, 2003 (Spain) | General population | Primary care centers | Cohort (R) | 1994-1999 | Hospitalization | 65+ | 30 | -67 | -192; 5 |
|  |  |  |  |  |  | 65-75 | NA | -143 | NA |
|  |  |  |  |  |  | 75+ | NA | -24 | NA |
|  | Pneumonia - no risk factors | Primary care centers | Cohort (R) | 1994-1999 | Hospitalization | 65+ | NA | 42 | NA |
| Tsai, 2015 (Taiwan) | General population | Health insurance database | Cohort (R) | 2009 | Hospitalization | 75+ | 8699 | 60 | 58; 60 |
| Christenson, 2004 (Sweden) | General population | Stockholm County | Cohort (P) | 1999-2000 | Hospitalization | 65+ | NA | 9 | 0; 18 |
| Skull, 2007 (Australia) | General population | Hospital | Case-cohort (R) | 2000-2002 | Hospitalization | 65+ |  | -1 | -16; 13 |
| Eurich, 2012 (Canada) | General population | CAP patients at emergency room | Cohort (P) (repeat CAP admission within 1 year) | 2000-2002 | Hospitalization | 18+ | NA | 9 | -20; 31 |
|  |  |  | Cohort (P) (repeat CAP admission within 5 years) | 2000-2002 | Hospitalization | 18+ | NA | -9 | -34; 11 |
| Ochoa-Gondar, 2014 (Spain) | General population | Primary care centers | Cohort (P) | 2008-2011 | Hospitalization | 60+ | 207 | 5 | -14; 20 |
| Vila-Corcoles, 2006 (Spain) | General population | Primary care centers | Cohort (P) | 2002-2005 | Outpatient | 65+ | 72 | 10 | -37; 41 |
| Jackson, 2003 (United States) | General population | Health maintenance organization | Cohort (R) | 1998-2001 | Outpatient | 65+ | NA | -4 | -13; 4 |
| *Immunocompromised* |  |  |  |  |  |  |  |  |  |
| Teshale, 2008 (United States) | HIV-infected patients | State and local health departments providing HIV care | Cohort (P) | 1998-2003 | Any severity or setting | 13+ | 1650 | 20 | 10; 20 |
|  | HIV-CD4 at vac: 0-99cell/ul | Health departments providing HIV care | Cohort (P) | 1998-2003 | Any severity or setting | 13+ | 242 | 20 | 10; 20 |
| Guerrero, 1999 (United States) | HIV-CD4 at vac: 0-99cell/ul | AIDS/HIV clinic (CDC ASD project) | Case-control (R) | 1989-1998 | Any severity or setting | 0+ | NA | 64 | 16; 84 |
| Teshale, 2008 (United States) | HIV-CD4 at vac: 100-199cell/ul | Health departments providing HIV care | Cohort (P) | 1998-2003 | Any severity or setting | 13+ | 246 | 20 | 10; 30 |
| Guerrero, 1999 (United States) | HIV-CD4 at vac: 100-199cell/ul | AIDS/HIV clinic (CDC ASD project) | Case-control (R) | 1989-1998 | Any severity or setting | 0+ | NA | 77 | 33; 92 |
|  | HIV-CD4 at vac: 200+cll/ul | AIDS/HIV clinic (CDC ASD project) | Case-control (R) | 1989-1998 | Any severity or setting | 0+ | NA | 78 | 46; 91 |
| Teshale, 2008 (United States) | HIV-CD4 at vac: 350-499cell/ul | Health departments providing HIV care | Cohort (P) | 1998-2003 | Any severity or setting | 13+ | 295 | 10 | -10; 20 |
|  | HIV-CD4 at vac: 500+cell/ul | Health departments providing HIV care | Cohort (P) | 1998-2003 | Any severity or setting | 13+ | 460 | 20 | 10; 30 |
|  | HIV-infected patients (time since vaccination 0-12 M) | Health departments providing HIV care | Cohort (P) | 1998-2003 | Any severity or setting | 13+ | 589 | 20 | 10; 20 |
|  | HIV-infected patients (time since vaccination 13-24 M) | Health departments providing HIV care | Cohort (P) | 1998-2003 | Any severity or setting | 13+ | 436 | 20 | 10; 20 |
|  | HIV-infected patients (time since vaccination 25-36 M) | Health departments providing HIV care | Cohort (P) | 1998-2003 | Any severity or setting | 13+ | 278 | 20 | 10; 30 |
|  | HIV-infected patients (time since vaccination 37-48 M) | Health departments providing HIV care | Cohort (P) | 1998-2003 | Any severity or setting | 13+ | 220 | 10 | -10; 30 |
|  | HIV-infected patients (time since vaccination 49-60 M) | Health departments providing HIV care | Cohort (P) | 1998-2003 | Any severity or setting | 13+ | 127 | 10 | -10; 30 |
| Hung, 2004 (Taiwan) | HIV-infected patients | Hospital providing HIV care | Cohort (P) | 2000-2002 | Any severity or setting | 15+ | 42 | -88 | -349; 21 |
| Lopez-Palomo, 2004 (Spain) | HIV-infected patients | Hospital providing HIV care | Cohort (P) | 1997-2000 | Any severity or setting | 18+ | 14 | 57 | 15; 79 |
| Guerrero, 1999 (United States) | HIV-infected patients | AIDS/HIV clinic | Case-control (R) | 1989-1998 | Any severity or setting | 0+ | 70 | 69 | 38; 84 |
| Lindenburg, 2001 (Netherlands) | HIV-infected patients | Amsterdam Cohort Study | Cohort (P&R) | ?-1998 | Any severity or setting | 0+ | 14 | -1 | -91; 47 |
| Rodriguez-Barradas,2008 (United States) | HIV-infected patients (male) | Veterans Affairs medical centers | Cohort (P) | 2001-2002 | Any severity or setting | 18+ | 50 | 35 | 0; 58 |
| Teshale, 2008 (United States) | HIV-ViralLoad at vac: 0-999cop/ml | Health departments providing HIV care | Cohort (P) | 1998-2003 | Any severity or setting | 13+ | 355 | 30 | 20; 40 |
|  | HIV-ViralLoad at vac: 10000-99999cop/ml | Health departments providing HIV care | Cohort (P) | 1998-2003 | Any severity or setting | 13+ | 370 | 10 | 0; 20 |
|  | HIV-ViralLoad at vac: 1000-9999cop/ml | Health departments providing HIV care | Cohort (P) | 1998-2003 | Any severity or setting | 13+ | 240 | 20 | 10; 30 |
|  | HIV-ViralLoad at vac:100000+cop/ml | Health departments providing HIV care | Cohort (P) | 1998-2003 | Any severity or setting | 13+ | 381 | 0 | -10; 10 |
| Curran, 2008 (Spain) | HIV-infected patients | Bacterial CAP patients admitted to hospital | Case-control (P) | 2000-2005 | High risk - severe pneumonia | 18+ | 4 | 60 | -40; 90 |
| Navin, 2000 (United States) | HIV-infected patients | CAP patients admitted to hospital | Case-control (P) | 1994-1996 | Hospitalization | 18+ | 34 | 32 | -15; 59 |
| Dominguez, 2010 (Spain) | Immunocompromised | Hospital | Case-control (P&R) | 2005-2007 | Hospitalization | 65+ | 130 | 21 | -19; 48 |
| *Underlying risk factors* |  |  |  |  |  |  |  |  |  |
| Ochoa-Gondar, 2008 (Spain) | Chronic respiratory disease | Primary care centers | Cohort (P) | 2002-2005 | Any severity or setting | 65+ | 107 | 23 | -7; 44 |
| Wagner, 2003 (Austria) | Geriatric hospital | Long-stay geriatric hospital | Case-control (R) | 1996-1998 | Any severity or setting | 65+ | 122 | 72 | NA |
| Chiou, 2015 (Taiwan) | Lung cancer | Health insurance database | Cohort (R) | 2007-2010 | Any severity or setting | 75+ | 79 | 26 | 2; 44 |
| Ochoa-Gondar, 2008 (Spain) | Chronic respiratory disease | Primary care centers | Cohort (P) | 2002-2005 | Death | 65+ | 14 | 13 | -128; 67 |
| Wagner, 2003 (Austria) | Geriatric hospital | Long-stay geriatric hospital | Case-control (R) | 1996-1998 | Death | 65+ | NA | 67 | NA |
| Ochoa-Gondar, 2008 (Spain) | Chronic respiratory disease | Primary care centers | Cohort (P) | 2002-2005 | Hospitalization | 65+ | 83 | 30 | 0; 52 |
| Nichol, 1999, AIM (United States) | Chronic respiratory disease | Managed care organization | Cohort (R) | 1993-1995 | Hospitalization | 65+ | NA | 43 | 16; 62 |
| Nichol, 1999, Vac (United States) | Chronic respiratory disease | Managed care organization | Cohort (R) | 1993-1996 | Hospitalization | 65+ | NA | 27 | -13; 52 |
| Hechter, 2012 (United States) | COPD population | Health maintenance organization | Cohort (P) | 2002-2009 | Hospitalization | 45-64 | NA | 1 | -41; 31 |
|  |  |  |  |  |  | 65+ | NA | -15 | -75; 25 |
| Vila-Corcoles, 2003 (Spain) | Pneumonia high risk | Primary care centers | Cohort (R) | 1994-1999 | Hospitalization | 65+ | NA | -43 | NA |
|  |  |  |  |  |  | 65-74 | NA | -338 | NA |
|  | Smokers | Primary care centers | Cohort (R) | 1994-1999 | Hospitalization | 65+ | 5 | 22 | -181; 78 |
| Hung, 2010 (China, Hong Kong) | Various risk factors | Outpatient clinics | Cohort (P) | 2007-2008 | Hospitalization | 65+ | NA | 23 | 7; 37 |
| Ochoa-Gondar, 2008 (Spain) | Chronic respiratory disease | Primary care centers | Cohort (P) | 2002-2005 | Outpatient | 65+ | 24 | -15 | -172; 52 |

CAP: community acquired pneumonia; CDC ASD project: Centers for Disease Control and Prevention Adult Spectrum of Disease project; CI: confidence interval; COPD: chronic obstructive pulmonary disease; HIV: human immunodeficiency virus; M: months; NA: not available; P: prospective; R: retrospective; VE: vaccine effectiveness
