# Supplementary material for: Effectiveness of pneumococcal vaccines in preventing pneumonia in adults, a systematic review and meta-analyses of observational studies
Source: PLoS One. 2017 May 23;12(5):e0177985. doi: 10.1371/journal.pone.0177985 (PMC5441633; doi:10.1371/journal.pone.0177985)
Supplement: S3 Table — (DOCX) [file pone.0177985.s009.docx]

**S3**

**Table S3.1. Results of the meta-regression: vaccine effectiveness against CAP requiring hospitalization.** Results of the full model.

| **Parameter** | **Regression coefficient** | **p-value** |
| --- | --- | --- |
| intercept | 19.4 | 0.179 |
| Age <65 yrs | -10.0 | 0.619 |
| Age >= 65 yrs | 2.6 | 0.855 |
| Design case-control | 4.0 | 0.681 |
| PCV in the private market | 28.3 | 0.004 |
| PCV in national immunization program | -0.5 | 0.972 |
| Maximum time since vaccination≥ 60 months | -26.9 | 0.022 |

**Table S3.2. Results of the meta-regression: vaccine effectiveness against CAP requiring hospitalization.** Results of the final model including maximum time since vaccination and type of PCV vaccination program as covariates.

| **Parameter** | **Regression coefficient** | **p-value** |
| --- | --- | --- |
| intercept | 22.9 | 0.001 |
| PCV in the private market | 28.3 | <0.001 |
| PCV in national immunization program | -1.9 | 0.839 |
| Maximum time since vaccination≥ 60 months | -27.7 | <0.001 |
