# Supplementary material for: Effectiveness of pneumococcal vaccines in preventing pneumonia in adults, a systematic review and meta-analyses of observational studies
Source: PLoS One. 2017 May 23;12(5):e0177985. doi: 10.1371/journal.pone.0177985 (PMC5441633; doi:10.1371/journal.pone.0177985)
Supplement: S4 Table — (DOCX) [file pone.0177985.s010.docx]

S4 Table. Study characteristics and results for pneumococcal CAP

| Author, year of publication (country) | Population | Population source | Study design | Study period | Clinical category | Age (years) | Cases (% bacteremic cases) | Vaccinated pCAP cases | VE(%) | 95%CI |
| --- | --- | --- | --- | --- | --- | --- | --- | --- | --- | --- |
| *General population* |  |  |  |  |  |  |  |  |  |  |
| Vila-Corcoles, 2009 (Spain) | General population | Primary care centers | Case-control (R) | 2002-2007 | Any setting or severity | 50+ | 304 (31%) | 112 | 48 | 27; 63 |
|  |  |  |  |  |  | 50-64 | 78 | 10 | 6 | -151; 65 |
|  |  |  |  |  |  | 65+ | 226 | 102 | 53 | 33; 68 |
|  |  |  |  |  |  | 65-79 | 135 | 59 | 48 | 19; 67 |
|  |  |  |  |  |  | 80+ | 91 | 43 | 56 | 16; 77 |
| Vila-Corcoles, 2006 (Spain) | General population | Primary care centers | Cohort (P) | 2002-2005 | Any setting or severity | 65+ | 70 (26%) | 32 | 45 | 12; 66 |
| Vila-Corcoles, 2009 (Spain) | General population - in influenza season | Primary care centers | Case-control (R) | 2002-2007 | Any setting or severity | 50+ | 165 (33%) | 57 | 60 | 27; 78 |
| Vila-Corcoles, 2006 (Spain) | General population - in influenza season | Primary care centers | Cohort (P) | 2005 | Any setting or severity | 65+ | 47 | 19 | 61 | 26; 79 |
| Vila-Corcoles, 2009 (Spain) | General population - out influenza season | Primary care centers | Case-control (R) | 2002-2007 | Any setting or severity | 50+ | 139 (29%) | 55 | 49 | 19; 69 |
| Vila-Corcoles, 2006 (Spain) | General population - out influenza season | Primary care centers | Cohort (P) | 2005 | Any setting or severity | 65+ | 23 | NA | 37 | -52; 74 |
| Wiemken, 2014 (Multi-country) | General population | CAP patients admitted to hospital (CAPO international cohort study database) | Case-control (R) | 2001-2012 | Hospitalization | 65+ | 325 | 46 | 37 | 16; 60 |
| Ochoa-Gondar, 2014 (Spain) | General population (exposed=PPV23 at any time; unexposed=never vaccinated) | Primary care centers | Cohort (P) | 2008-2011 | Hospitalization | 60+ | 113 (11%) | NA | 32 | -18; 61 |
| Ochoa-Gondar, 2014 (Spain) | General population (exposed=PPV23 5y before study start; unexposed=never vaccinated) | Primary care centers | Cohort (P) | 2008-2011 | Hospitalization | 60+ | 84 (14%) | 45 | 51 | 16; 71 |
| *Immunocompromised* |  |  |  |  |  |  |  |  |  |  |
| Lopez-Palomo, 2004 (Spain) | HIV-infected patients | Hospital providing HIV care | Cohort (P) | 1997-2000 | Any severity or setting | 18+ | 12 (58%) | 3 | 77 | -6; 92 |
| Vila-Corcoles, 2009 (Spain) | Pneumonia risk - Stratum 1* | Primary care centers | Case-control (R) | 2002-2007 | Any severity or setting | 50+ | 64 | 25 | 71 | 24; 89 |
|  | Pneumonia risk -Stratum 2-3* | Primary care centers | Case-control (R) | 2002-2007 |  | 50+ | 240 | 87 | 45 | 20; 62 |
| *Underlying risk factors* |  |  |  |  |  |  |  |  |  |  |
| Vila-Corcoles, 2012 (Spain) | Chronic respiratory disease | Primary care centers | Case-control (R) | 2002-2007 | Any severity or setting | 50+ | 96 (20%) | 58 | 29 | -39; 63 |
|  | Chronic respiratory disease | Primary care centers | Case-control (R) |  |  | 50-74 | 51 | 31 | -48 | -256; 38 |
|  | Chronic respiratory disease | Primary care centers | Case-control (R) |  |  | 75+ | 45 | 27 | 55 | -27; 84 |
|  | Chronic respiratory disease - in influenza season | Primary care centers | Case-control (R) |  |  | 50+ | 54 | 33 | 44 | -41; 78 |
|  | Chronic respiratory disease - out influenza season | Primary care centers | Case-control (R) |  |  | 50+ | 42 | 25 | -7 | -153; 55 |
| Vila-Corcoles, 2009 (Spain) | Pneumonia risk -Stratum 2* | Primary care centers | Case-control (R) | 2002-2007 | Any severity or setting | 50+ | 172 | 74 | 41 | 10; 61 |
|  | Pneumonia risk -Stratum 3* | Primary care centers | Case-control (R) |  |  | 50+ | 68 | 13 | 61 | -2; 85 |
| Ochoa-Gondar, 2008 (Spain) | Chronic respiratory disease | Primary care centers | Cohort (P) | 2002-2005 | Hospitalization | 65+ | 20 | 12 | 24 | -90; 70 |
| Hung, 2010 (China, Hong Kong) | Various risk factors | Outpatient clinics | Cohort (P) | 2007-2008 | Hospitalization | 65+ |  | NA | 38 | -5; 70 |

CI: confidence interval; NA: not available; P: prospective; pCAP: pneumococcal pneumonia; py: person-years; R: retrospective; VE: vaccine effectiveness
*Stratum 1: persons with conditions associated with possible immunocompromise: immunodeficiency (including AIDS), asplenia, cancer (solid organ orhaematological neoplasia), chronic nephropathy (nephrotic syndrome, renal failure, dialysis or transplantation), and long-term corticosteroid therapy (20 mg/day of prednisone or equivalent). Stratum 2: patients without a level 1 condition (i.e. possible immunocompromised) but who had a history of chronic lung disease (chronic bronchitis, emphysema or asthma), liver disease (cirrhosis or alcoholic hepatitis), heart disease (congestive heart failure or chronic angina) and diabetes mellitus. Stratum 3: patients without a level 1 (i.e. possible immunocompromised) or level 2 (see definition of stratum 2 above) condition.
