# Supplementary material for: Effectiveness of pneumococcal vaccines in preventing pneumonia in adults, a systematic review and meta-analyses of observational studies
Source: PLoS One. 2017 May 23;12(5):e0177985. doi: 10.1371/journal.pone.0177985 (PMC5441633; doi:10.1371/journal.pone.0177985)
Supplement: S5 Table — (DOCX) [file pone.0177985.s011.docx]

S5 Table. Study characteristics and results for nonbacteremic pneumococcal CAP

| Author, year of publication (country) | Population | Population source | Study design (P/R) | Study period | Heathcare setting | Age | Vaccinated nonbacteremic pCAP cases | VE | 95%CI |
| --- | --- | --- | --- | --- | --- | --- | --- | --- | --- |
| *General population* |  |  |  |  |  |  |  |  |  |
| Vila-Corcoles, 2009 (Spain) | General population | Primary care centers | Case-control (R) | 2002 - 2007 | Any setting or severity | 50+ | 82 | 42 | 14; 61 |
| Vila-Corcoles, 2006 (Spain) | General population | Primary care centers | Cohort (P) | 2002 - 2005 | Any setting or severity | 65+ | 26 | 39 | -6; 65 |
| Musher, 2006 (United States) | General population | Patients with *S. pneumonia* | Case-control (R) | 2001 - 2005 | Any setting or severity | 18+ | 49 | -1 | -86; 45 |
| Vila-Corcoles, 2009 (Spain) | General population - in flu season | Primary care centers | Case-control (R) | 2002 - 2007 | Any setting or severity | 50+ | 42 | 54 | 6; 77 |
|  | General population - out flu season | Primary care centers | Case-control (R) | 2002 - 2007 | Any setting or severity | 50+ | 40 | 44 | 3; 68 |
| Ochoa-Gondar, 2014 (Spain) | General population | Primary care centers | Cohort (P) | 2008 - 2011 | Hospitalization | 60+ | 42 | -3 | -53; 31 |
| *Underlying risk factors* |  |  |  |  |  |  |  |  |  |
| Vila-Corcoles, 2012 (Spain) | Chronic respiratory disease | Primary care centers | Case-control (R) | 2002- 2007 | Any severity or setting | 50+ | 46 | 34 | -34; 67 |

CI: confidence interval; P: prospective; pCAP: pneumococcal community-acquired pneumonia; R: retrospective; VE: vaccine effectiveness
